# Contact-Based Methods for Measuring Respiratory Rate

**DOI:** 10.3390/s19040908

**Published:** 2019-02-21

**Authors:** Carlo Massaroni, Andrea Nicolò, Daniela Lo Presti, Massimo Sacchetti, Sergio Silvestri, Emiliano Schena

**Affiliations:** 1 Unit of Measurements and Biomedical Instrumentation, Department of Engineering, Università Campus Bio-Medico di Roma, Via Alvaro del Portillo, 21, 00128 Rome, Italy; d.lopresti@unicampus.it (D.L.P.); s.silvestri@unicampus.it (S.S.); e.schena@unicampus.it (E.S.); 2 Department of Movement, Human and Health Sciences, University of Rome “Foro Italico”, 00135 Rome, Italy; andrea.nicolo@yahoo.com (A.N.); massimo.sacchetti@uniroma4.it (M.S.)

**Keywords:** sensors, respiratory frequency, measurement, wearable, contact-based

## Abstract

There is an ever-growing demand for measuring respiratory variables during a variety of applications, including monitoring in clinical and occupational settings, and during sporting activities and exercise. Special attention is devoted to the monitoring of respiratory rate because it is a vital sign, which responds to a variety of stressors. There are different methods for measuring respiratory rate, which can be classed as contact-based or contactless. The present paper provides an overview of the currently available contact-based methods for measuring respiratory rate. For these methods, the sensing element (or part of the instrument containing it) is attached to the subject’s body. Methods based upon the recording of respiratory airflow, sounds, air temperature, air humidity, air components, chest wall movements, and modulation of the cardiac activity are presented. Working principles, metrological characteristics, and applications in the respiratory monitoring field are presented to explore potential development and applicability for each method.

## 1. Introduction

There is an ever-growing demand for measuring respiratory variables during a variety of applications. Respiratory rate, also known as respiratory frequency ($f_{R}$), appears to be among the most promising and measured variables because it provides fundamental information. In view of its peculiar neurophysiological regulation, $f_{R}$ is very sensitive to a variety of physiological, psychological and environmental stressors [1,2,3], and is considered to be one of the most informative vital signs [4,5]. Conversely, tidal volume (the other determinant of minute ventilation) plays a major role in satisfying the metabolic requirements of the human body [1,2,3]. This explains why $f_{R}$ and tidal volume have been defined as the behavioral and metabolic components of minute ventilation, respectively [1]. The differential control of $f_{R}$ and tidal volume justifies the special attention devoted to $f_{R}$ monitoring.

There are different methods for measuring $f_{R}$, which complicates the choice of the specific sensor or measurement technique to use. Several factors need to be considered when measuring $f_{R}$, including applications, measurement requirements, and user needs. This choice is even more difficult in view of the limited attempts made so far to classify and detail the numerous $f_{R}$ measuring methods used [6,7,8]. Therefore, we aim to provide an overview of the currently available methods for measuring $f_{R}$. Given the extent of the topic, this review focuses on the contact-based methods only, to guarantee that each method is described in sufficient detail to enable the reader to make an informed choice on the $f_{R}$ method to use.

The paper is structured in nine sections. Section 1 provides a brief description of the importance of monitoring $f_{R}$ in clinical settings, occupational settings, and during sporting activities and exercise. Besides, Section 1 provides a taxonomy of the available techniques for measuring $f_{R}$, which are categorized according to the measurand. For each of the methods identified in the taxonomy, we have dedicated a separate Section (from Section 2, Section 3, Section 4, Section 5, Section 6, Section 7 and Section 8), which consists of a brief introduction of the measuring principle and a short overview of the most popular sensors that can be used to transduce the physical or chemical quantity into a signal to extract $f_{R}$. At the end of each section, a table summarizes metrological properties, sensor characteristics and possible applications, and a short summary describes strengths and weaknesses of the different sensors. Specifically, we describe methods based on airflow (Section 2), respiratory sounds (Section 3), air temperature (Section 4), air humidity (Section 5), air components (Section 6), respiratory-induced chest wall movements (Section 7), and respiratory modulation of cardiac activity (Section 8). The last section is dedicated to conclusions (Section 9).

### 1.1. The Importance of Respiratory Rate Monitoring

Before dealing with the $f_{R}$ methods, we briefly describe the major applications where the monitoring of $f_{R}$ is invaluable. A further aim is to increase awareness of the importance of measuring $f_{R}$. Indeed, despite a large body of evidence indicating that $f_{R}$ is a very relevant vital sign that responds to a variety of stressors, this parameter has long been overlooked in the clinical field [4,9] as well as in other fields [8].

#### 1.1.1. Clinical Settings

Substantial evidence indicates that $f_{R}$ is a very informative vital sign. It is an early indicator of physiological deterioration [9,10] and a predictor of potentially serious adverse events [4]. Specifically, $f_{R}$ is an important predictor of cardiac arrest and of intensive care unit admission [4] as well as an independent prognostic marker for risk assessment after acute myocardial infarction [11]. Besides, it is fundamental in the early detection of the risk of the occurrence of dangerous conditions such as sleep apnea [12], respiratory depression in post-surgical patients [13] and sudden infant death syndrome [14]. Furthermore, $f_{R}$ is sensitive to several other pathological conditions including diabetic ketoacidosis, toxicological issues, shock, pain, sepsis, allergic reactions and dehydration [5]. Yet, $f_{R}$ is overlooked and under-recorded [4]. Suffice to consider that among four classic vital signs, $f_{R}$ is the only one that is often measured clinically instead of with objective devices [5], and is frequently omitted from hospital documentation [4]. The limited consideration given to $f_{R}$ is partially explained by the fact that there are no clear indications on how $f_{R}$ should be measured in clinical settings, and that clinical staff has low confidence in the methods currently used to assess $f_{R}$ [15]. Therefore, technological development and validation studies [16] are required to fill the gap between the urgent need to accurately measure $f_{R}$ in clinical settings and current clinical practice. The present review may help to reduce this gap.

#### 1.1.2. Occupational Settings

Technological development is driving an increasing interest in the monitoring of workers during their activities, with the aim to improve health, well-being, and safety. Various multiparameter wearable devices measuring $f_{R}$ have been developed and tested with the aim of monitoring workers facing high levels of psychophysiological stress [17,18]. Monitoring $f_{R}$ during working activities is of great value because $f_{R}$ is sensitive to cognitive load, emotional stress, environmental challenges, pain, and discomfort, among other factors [3,19]. Specifically, $f_{R}$ has been proposed as a sensitive marker of cognitive load, with important implications for workers exposed to highly demanding tasks and weighty responsibilities, including pilots, soldiers and surgeons [19,20]. Besides, $f_{R}$ can be used as an indicator of thermal stress because it is substantially influenced by body temperature [21]. This is important for workers operating in hot environments as well as for those wearing protective clothing and equipment that may interfere with thermoregulation, including firefighters [22].

#### 1.1.3. Sport and Exercise

The importance of measuring $f_{R}$ during exercise has recently been reviewed by Nicolò et al. [8]. The authors make a point for $f_{R}$ being a better marker of physical effort compared to traditionally monitored physiological variables such as oxygen uptake, blood lactate and heart rate [8]. Unlike these variables, $f_{R}$ is closely associated with perceived exertion in a variety of exercise conditions [2,23,24,25] and experimental interventions [8], and responds very rapidly to abrupt changes in work rate which occur during intermittent exercise [8,25,26]. This is important because many sporting activities are of intermittent nature, including soccer and other team sports. Measuring $f_{R}$ during exercise is not only relevant for athletes but also for other populations. For instance, $f_{R}$ is associated with exercise-induced dyspnea [27] and is a marker of exercise tolerance in patients with chronic obstructive pulmonary disease [28]. Furthermore, $f_{R}$ can provide information on physical effort in cardiopathic patients who have heart rate altered by pharmacological interventions such as beta blockers.

However, the measure of $f_{R}$ during exercise has been neglected so far [8], and there is a paucity of respiratory wearable devices that have been specifically designed for sporting activities. Yet, different exercise modalities may introduce diverse methodological challenges that need to be faced, with motion artifacts being a classical example. Therefore, a detailed overview of the contact-based techniques for measuring $f_{R}$ may favor the development and use of respiratory devices designed for specific applications, including the monitoring of $f_{R}$ during exercise.

### 1.2. Taxonomy of Available Techniques for Respiratory Rate Monitoring

**Figure 1:**
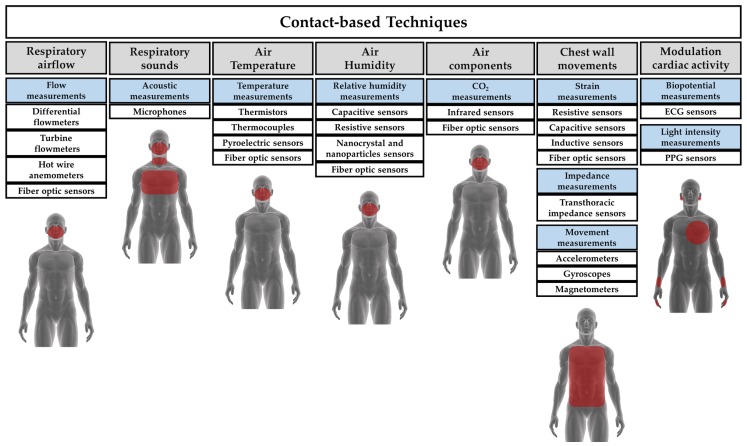
Most popular contact-based techniques for measuring $f_{R}$ and related areas of the body on which the sensors must be positioned. PPG = photoplethysmography; ECG = Electrocardiography.

Different technologies are available for measuring $f_{R}$. Such technologies may be classed in contact-based and contactless. In contact-based measuring techniques, the sensor (i.e., the element directly affected by the measurand) must be in contact with the subject’s body. Figure 1 shows a classification of the most popular contact-based techniques for $f_{R}$ monitoring and the related area of the body where the sensor must be positioned. In this review, we provide an overview of sensors and measuring systems with different working principles, based on physical and chemical principles. We have identified seven different techniques, categorized according to the measurand. Some of these techniques have been split into subclasses: it is the case of chest wall movement sensors that can be classed in sensors devoted to transducing chest wall strains, impedance changes, and tridimensional motion caused by the respiratory activity. Similarly, several techniques can be used to retrieve $f_{R}$ values and respiratory activity from the modulatory effect that breathing has on cardiac activity (e.g., biopotential and light intensity modulation). In this review, the Table at the end of each section provides a qualitative overview of:

(*i*) metrological properties:

- sensitivity, according to the definition reported in the International Vocabulary of Metrology [29];
- step response time, according to [29];
- output linearity, according to [29];
- accuracy, according to [29].

(*ii*) sensor characteristics:

- Size (i.e., the size of the sensor used to collect the physical/chemical quantity);
- Cost (including an estimate of the cost of signal conditioning electronics);
- Real-time monitoring: ability to record the respiratory signal (and $f_{R}$ values) in real time;
- Measurement intrusiveness: how the sensor or the measuring technique limits the subject’s activity and movements;
- Sensitivity to body motion artifacts: sensitivity of a measuring technique to movements and motions not related to breathing that negatively affect the output signal;
- Influence of environmental factors: influence of temperature, humidity, external strains and other environmental factors that can affect sensor measurement and consequently the sensor output;
- Presence of wire: presence of tube, wires, and connections needed to supply the sensors, and/or register the physical/chemical quantity, and/or transfer the data for processing.

(*iii*) possible applications based on the aforementioned properties and characteristics. The typical values of $f_{R}$ at rest measured in breaths per minute (in short bpm) vary with age. In this review, we have considered the following applications related to specific breathing ranges (valid for adults with age < 65 years old [30]):

- slow breathing (in short SB) [31]: $f_{R}$ < 12 bpm
- quiet breathing (in short QB) [31,32]: 12 bpm ≤ $f_{R}$ ≤ 20 bpm;
- fast breathing (in short FB) [31]: $f_{R}$ ≥ 21 bpm

While a $f_{R}$ ≥ 21 bpm is classified as FB at rest, values higher than 60 bpm are commonly registered during high-intensity exercise [8]. Therefore, when possible, we provide more specific information on the highest $f_{R}$ that can be registered with different sensors.

## 2. Techniques Based on Respiratory Airflow

**Figure 2:**
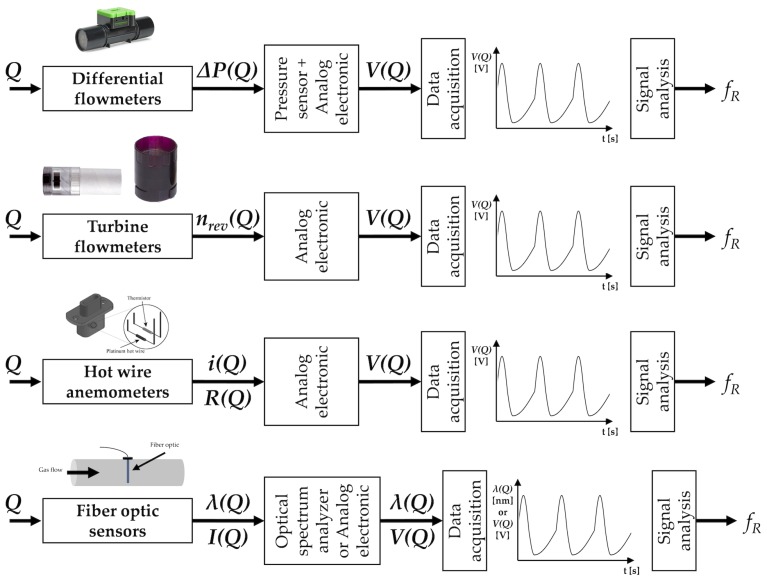
Airflow sensors: the main elements composing the measuring chain (sensor, analog electronics, data acquisition, post-processing or signal analysis stage) to estimate $f_{R}$. Analog electronic stages vary between different flowmeters because the output quantities are different. ΔP(Q), $n_{rev}\left(Q\right)$, i(Q), R(Q), λ(Q), I(Q) are pressure drop, turbine revolutions per minute, current, resistance, wavelength, and light intensity changes caused by the airflow (*Q*), respectively. V(Q) is the voltage output. The DF figure is from Sensirion [33]; the turbine flowmeter figure is from MIR [34]; the HWA figure is from [35].

Different sensors can be used for measuring the volume and/or the velocity of the inhaled and exhaled air during breathing. The temporal trend of volume or velocity allows to estimate $f_{R}$. Among others, differential flowmeters (DFs), turbine flowmeters, hot wire anemometers (HWAs) and fiber-optic-based flowmeters have been investigated (see Figure 2).

### 2.1. Flow Sensors

#### 2.1.1. Differential Flowmeters

DFs are largely used for collecting the temporal trend of inhaled and exhaled air during breathing. In addition, DFs have gained broad acceptance as sensors for monitoring gases delivered by mechanical ventilators and recorded by commercial spirometers [36,37]. This kind of flowmeters consists of a pneumatic resistance placed within the pipe in which the gas flows. This resistance transduces the gas flowrate (*Q*) within a pressure drop (ΔP) with a well-known relationship. Accordingly, these flowmeters need a secondary element which measures the ΔP value (i.e., a differential pressure sensor). There are two main types of DFs used in the field of interest:

1. *Pneumotachographs*. They can be subdivided into Fleisch, where the resistance consists of capillary tubes [38], and into Lilly, where the resistance is a fine wire mesh [39]. In both cases, Hagen-Poiseuille law may express the linear relationship between the output (ΔP) and the input (*Q*):
  (1) $$\Delta P=\frac{8\cdot \mu \cdot L}{n\cdot \pi \cdot r^{4}}\cdot Q$$
  being μ the dynamic viscosity of the gas, and *L*, *r*, and *n* the length, the radius, and the number of the capillary, respectively. Although quadratic models have been investigated [40,41], the linear relationship (Equation (1)) is mostly used for pneumotachographs. Linearity and a good frequency response (response time in the order of tens of ms) are two key factors for the use of this flowmeter in respiratory monitoring, if an adequate differential pressure sensor is chosen. One potential concern is related to the influence of the gas composition and the temperature on the pneumotachograph response because these factors affect the value of μ in Equation (1). Nevertheless, this aspect is relevant for accurate flow measurements, while it can be neglected for the estimation of $f_{R}$.
2. *Orifice meters*. They can be split into fixed orifice meters, where the resistance is an orifice plate, and into variable orifice meters, where the plate composing the resistance increases its passage area with flowrate (e.g., it consists of a flexible flap [42,43]). In both cases, the input-output relationship (ΔP vs. *Q*) may be expressed as follows:
  (2) $$Q_{i}=\frac{d^{2}}{\sqrt{1-\beta^{4}}}\frac{\sqrt{2\cdot \Delta P}}{\rho }$$
  where $Q_{i}$ is the flowrate calculated considering ideal conditions, *d* is the diameter of the orifice, β the ratio between the diameter of the orifice and the internal diameter of the pipe, and ρ the gas density. Therefore, the input-output relationship is not linear for fixed orifice meter; conversely, the increase of the passage area with flow in the variable orifice meter has the effect of linearizing the input-output relationship. As for pneumotachographs, they have a good frequency response when an adequate differential pressure sensor is chosen.

DFs are used to monitor the trend of respiratory flows in both adults and infants since they are designed to reach an ideal trade-off between sensitivity and additional resistance to the respiratory system. Indeed, an adequate design of the resistance for both pneumotachographs and orifice meters can adjust the sensitivity and the measuring range of these sensors allowing the development of sensors able to either measure very low flowrates (<0.1 L·min^−1^) or to have a wide range of measurements (>100 L·min^−1^). In addition, DFs can discriminate between inhaled and exhaled flows since they are bidirectional. However, the need to collect the entire inhaled and exhaled airflow may pose an important problem because further resistance is added, and the presence of a collector (e.g., a face-mask) may sometimes not be well tolerated by critically ill patients or even athletes [6]. These flowmeters are largely used for respiratory monitoring. For instance, this kind of flowmeter is embedded in medical devices (e.g., spirometers and mechanical ventilators). They are often used as reference tools to assess new techniques designed ad hoc for $f_{R}$ (or respiratory function) monitoring [44,45,46] because they measure the flow directly.

#### 2.1.2. Turbine Flowmeters

Similar to DFs, turbine flowmeters are used to collect respiratory temporal trends during breathing. Several spirometers and metabolic carts embed this type of sensors. These flowmeters consist of a turbine with flat blades in which the air ventilated by the patient is conveyed externally to the distributor. The distributor is represented by a cylinder with slots on its surface. Slots orient the air flow tangentially with respect to the blades [47]. The dimensional analysis of this turbine provides the following equation:

(3) $$\frac{Q}{n_{rev}\cdot D^{3}}=f\left(\frac{n_{rev}\cdot D^{2}}{\nu }\right)$$

where $n_{rev}$ is the turbine revolutions per minute, *D* is the inner diameter of the distributor, and ν is the kinematic viscosity. The function $\frac{Q}{n_{rev}\cdot D^{3}}$ can be obtained as a function of $\frac{n_{rev}\cdot D^{2}}{\nu }$ (that is the number of Reynolds), when ν is negligible. The mass of the blades is tens of mg, and this allows to obtain a reduced moment of inertia with respect to the rotation axis. For this reason, the dynamic response is excellent with a rise time much shorter than 10 ms [47]. The number of revolutions is typically collected using a phototransistor that records the passage of the blade in front of it, providing an impulse for each passage. The higher is the number of revolutions per minute, the higher is the *Q* in accordance with the following Equation (4):

(4) $$Q=k\cdot n_{rev}$$

For Reynolds number values greater than 800–1000, the ratio $\frac{Q}{n\cdot D^{3}}$ is constant: the relationship between *Q* and $n_{rev}$ is linear, independent from fluid properties (e.g., ν) but dependent on the geometry of the sensor [47]. An adequate design of the mechanical parts of the sensors can adjust their sensitivity and the measuring range to allow the linear relationship in Equation (4) to be valid also at low *Q* values (≤1 L·min^−1^). These sensors do not need any correction for temperature on exhalation and are not affected by humidity or altitude changes [48]. Turbine flowmeters can be placed at the end of a mouthpiece in which the patient can breathe (with the nose closed by a nose clip) or they can be positioned at the distal extremity of a face-mask.

Turbine flowmeters are used to measure the breathing pattern in the clinical practice [48] and to extract respiratory variables and parameters (e.g., the $f_{R}$, tidal volume, FEV1 and FVC) [49]. These sensors show good performance in the estimation of the duration of inspiratory and expiratory breathing phases, and of the respiratory period [50]. Turbine flowmeters are the most used sensors in commercial certified portable spirometers (medical devices), and they are usually used to provide reference measurements for the assessment of new measuring systems. In the field of exercise science, most companies selling metabolic carts (e.g., COSMED S.r.l., Rome, Italy) adopt these flowmeters in their instruments [51]. The validity of turbine flowmeters has been extensively demonstrated for a wide range of $f_{R}$ values and during various exercise modalities of different intensities [8,52,53], suggesting that turbine flowmeters are not affected by motion artifacts.

#### 2.1.3. Hot Wire Anemometers

Similar to DFs and turbine flowmeters, HWAs are used for collecting temporal trends of inhaled and exhaled air during breathing, and they are embedded in several commercial mechanical ventilators and spirometers [36]. These flowmeters consist of one or more heated wires exchanging heat with the fluid flow. The equilibrium temperature of the wire ($T_{W}$) can be expressed as [54]:

(5) $$i^{2}\cdot R_{W}=h\cdot S\cdot \left(T_{W}-T_{g}\right)$$

where *i* is the current flowing through the wire, $R_{W}$ is the wire resistance, *h* is the coefficient of heat transfer, *S* is the exchange surface, and $T_{g}$ is the temperature of the gas. The King’s law expresses the relationship between *h* and the velocity of the gas (*v*) that hits the wire [55]:

(6) $$h=A+B\cdot \sqrt{\left(\rho \cdot v\right)}$$

where *A* and *B* are two empirical constants.

As shown by Equations (5) and (6), HWAs have a nonlinear response with high sensitivity at low *Q* values; hence they are mainly used for pediatric applications where *Q* is low. Due to the small size of the wire (typical diameter of ∼10 µm), the dynamic response is excellent with a rise time much shorter than 1 ms allowing a flat frequency response up to hundreds of Hz. The main concern for the use of HWAs in $f_{R}$ monitoring is related to their fragility due to the small size of the wire. Moreover, the typical configuration with a single wire is not able to discriminate the direction of the flow, hence the use of more complex configurations based on at least two wires are used to discriminate between inspiratory and expiratory phases [56]. HWAs are used to measure the breathing pattern in clinical settings, showing good performances in the estimation of the duration of inspiratory and expiratory breathing phases, and of the $f_{R}$. They are embedded in several commercial medical devices (mechanical ventilators and spirometers), and they are also used to provide reference measurements for the assessment of new systems [57,58,59]. They are often used with face-masks, but other solutions have also been proposed [60]. For instance, a tube-free configuration has recently been proposed as it allows non-intrusive respiratory monitoring [61].

#### 2.1.4. Fiber-Optic Based Flowmeters

Different types of fiber-optic sensors (FOSs) have recently been proposed to monitor flowrates. Among others, intensity-based FOSs, and fiber Bragg grating (FBG) sensors have been investigated [62,63,64,65,66,67]. Intensity-based FOSs have been proposed for breathing pattern monitoring [62,68,69,70]. The working principle of the flowmeters proposed in these articles is based on the changes of coupled light power with a fiber bending caused by the inhaled or exhaled airflow. Therefore, the light power measured by a photodetector represents the sensor output and depends on *Q*. The high sensitivity allows detecting low airflow (<1 L·min^−1^) [71]. Custom configurations allow the use of these sensors even in harsh environments and in the presence of strong electromagnetic fields (e.g., during magnetic resonance scans), where electronic sensors cannot be used. The sensors proposed in [68,69] were also tested on humans proving their ability to measure the airflow, but no comparison was made with a reference system to assess the accuracy of the sensors. The intensity-based sensor proposed in [69] may help distinguish between different breathing conditions (e.g., regular and irregular breathing, SB, QB and FB patterns). The sensing element of these sensors may be considered as a small-sized fiber-optic (diameter of hundreds of µm). The metrological properties depend on specific configuration used, and sensors with good static and dynamic performance may be potentially designed.

Table 1 summarizes the main metrological properties, sensor characteristics, and applications for each airflow sensor.

### 2.2. Short Summary

Flowmeters are used for respiratory activities monitoring because of their accuracy, sensitivity and frequency response especially in the case of DFs, turbine flowmeters, and HWAs. These sensors must be exposed to the air inhaled and exhaled by the subject. The consequence is that their measurement is usually quite intrusive, although the small size of the sensing element in the case of turbine flowmeters, HWAs and of fiber-optic-based flowmeters allows designing configurations that permit unobstructed measurements. Because of the direct measurement of *Q*, flowmeters are often used as a reference tool to assess new techniques designed ad hoc for $f_{R}$ monitoring (even on breath-by-breath basis). When all the expired and inhaled flow is conveyed into the flowmeters, these sensors allow recording breathing lung volume and tracking its changes. One of the main concerns related to the use of HWAs is their fragility, while the DFs are more solid. Turbine flowmeters are good in a wide range of *Q*, and their performance is unaffected by humidity, temperature, or altitude changes. Finally, fiber-optic-based flowmeters may potentially have several advantages (small size, short response time, usability in harsh environments) but their use is still at a relatively early stage. The first application in respiratory monitoring on subjects was in 2010, even though no detailed description of their performance can be found in literature.

## 3. Techniques Based on Respiratory Sounds

During breathing, the sound generated by the air flowing through the patient’s throat and airways may be collected to investigate the inspiration and expiration phases of breathing to estimate $f_{R}$ [72]. The respiratory sounds are characterized by broad spectrum noise with a frequency range depending on the body location of the sensor (i.e., mouth, trachea or chest wall) [72]. Acoustic sensors adhering to the skin can be used to measure these sounds. All the measuring techniques and related algorithms can be referred to Computerized Respiratory Sound Analysis (CORSA). Standardization of CORSA have recently been provided along with guidelines for breath sound recording [72].

**Figure 3:**
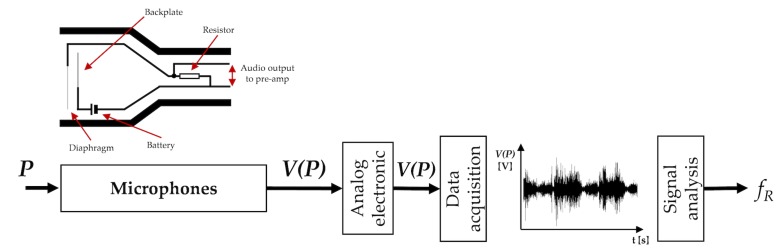
Acoustic sensors: the main elements composing the measuring chain (sensor, analog electronics, data acquisition, post-processing or signal analysis stage) to estimate $f_{R}$. *P* is the acoustical pressure; V(P) is the voltage output. The acoustic sensor scheme is adapted from [74].

The working principle of acoustic sensors is based on their sensitivity to physical changes of the environment surrounding the sound source. The most common sensors are microphones used to record air pressure changes caused by the sound waves (see Figure 3) [73].

### 3.1. Acoustic Sensors

#### Microphones

A microphone is a transducer that converts acoustic pressure variation into an electrical signal. Different methods may be employed to accomplish this conversion: capacitors, electrets, moving-coils, piezoelectric elements and fiber optics can be used as primary elements of the measuring chain [75].

The most common primary methods of transduction employ capacitors. In the capacitor microphones, one plate of the condenser is fixed while the other plate (i.e., the diaphragm), which is very close to it, is free to vibrate when the sound strikes it [76]. As diaphragm moves in and out under the influence of sound waves, the capacitance varies following Equation (7):

(7) $$Q_{E}=C\cdot E$$

where $Q_{E}$ is the electrical charge on the plates, *C* is the capacitance, and *E* is the applied voltage (across a variable capacitance). Typically, a battery is used to establish the charge to the condenser; pre-polarized condenser elements (known as electrets) may also be alternatively used [76]. Additional circuitry may be used to reduce electrical noise and to achieve wide dynamic ranges.

Dynamic microphones use the principle of magnetic induction in which a coil of wire produces a small output voltage as it moves through a magnetic field. The voice coil is generally no larger than 1 cm in diameter to cover the required audio frequency range. The voice coil is attached to a light diaphragm (in aluminum or plastic) [76]. Dynamic microphones are relatively unaffected by temperature or humidity.

Usually, the output provided by microphones is amplified, filtered in the bandwidth of 50–2500 Hz and digitized by a sampling rate higher than at least 5 kHz [77].

During the inspiratory and expiratory phases, the relationship between tracheal sound amplitude and flow can be represented with a power law curve [78]. Generally, the sound is recorded over the suprasternal notch of the trachea since this part of the upper airways is generally not affected by lung disease. The tracheal sound has a direct relationship with airflow and reaches frequency values up to 1500 Hz under physiological conditions [77]. Alternatively, normal breath sounds can be recorded at the level of the upper anterior chest wall. These breath sounds have approximately similar intensities during the inspiratory and expiratory phases of breathing. When the microphone is positioned on the posterior chest wall, abnormal breathing sounds may be identified because they contain higher-frequency components and a higher intensity than that of normal breathing sounds [72].

The estimation of $f_{R}$ using an acoustical approach has recently gained popularity in clinical settings. Different computational techniques can be used to obtain breathing information, as detailed in [79]. $f_{R}$ can be monitored at the bedside using an adhesive sensor with an integrated acoustic transducer positioned on the patient’s throat, as in [80]. When the adhesive sensor was compared to a CO_2_ sensor used as a reference instrument, a bias of ∼0.0 ± 1.4 bpm was found, with $f_{R}$ ranging from 6 to 24 bpm [80] (hereinafter the bias is reported as MOD ± LOA, obtained from Bland-Altman analysis [81]. The bias was used to provide a quantitative values of the agreement between $f_{R}$ estimated by the technique and reference values. MOD = mean of the differences; LOAs = limits of agreement, calculated as 1.96 times the standard deviation of the $f_{R}$ measurements estimated by the two methods).

In [82] sounds were acquired in the same position and analyzed using a combined investigation of the sound envelope and frequency content. Recently, a commercial device that monitors $f_{R}$ via tracheal sounds has been introduced in clinical settings (Masimo Rainbow SET Acoustic Monitoring, Masimo Corp., Irvine, CA, USA). However, only a few attempts have been made to develop portable and unobtrusive devices measuring $f_{R}$ outside the clinical setting [83]. For instance, a miniaturized and wearable system to estimate $f_{R}$ composed by a small sensor and a noise-robust algorithm has been described in [83]. Good results have also been obtained using microphones embedded into a smartphone; when compared to reference measurements (i.e., by a spirometer), a bias of 0.0 ± 1.5 bpm was found for subjects breathing in a quiet room, with $f_{R}$ ranging from 15 to 35 bpm [78]. Using the microphone of a Bluetooth headset (breath sounds were recorded at the level of the mouth), breath-by-breath monitoring of $f_{R}$ was carried out during running activity in an outdoor scenario by applying specific filters and a sound training phase to discriminate breath content from noise [84]. However, acoustical approaches remain extremely susceptible to background noise (i.e., music, traffic, other people speech) and to subject activities not related to the breathing such as arms/legs movements, speaking, coughing and swallowing. Furthermore, an appropriate placement of the sensor is critical to obtaining accurate and reliable monitoring with this method [80], especially outside the clinical scenario. Additionally, the actual $f_{R}$ estimation by acoustical means requires many more signal processing techniques [84,85] and investigations (i.e., heart sound cancellation [77], body vibration/movement cancellation or attenuation [86]) than other techniques (e.g., based on the air flow or temperature measurements). For these reasons, this technique does not usually allow real-time monitoring of $f_{R}$.

Table 2 summarizes the main metrological properties, sensors characteristics and applications of the acoustic sensors used for $f_{R}$ monitoring.

### 3.2. Short Summary

Microphone sensors are becoming of interest to respiratory activity monitoring because of their reduced encumbrance and because they can be embedded in wearable devices. However, acoustical approaches remain susceptible to both background noise and subject activity not related to the breathing. This measuring method is not generally recommended to record the respiratory waveform over time. Some studies have demonstrated good performance in extremely structured environments (i.e., intensive care unit). A few studies have been investigated this technique outside the clinical scenario (i.e., sporting activities), but they lack preliminary validation studies.

## 4. Techniques Based on Air Temperature

During breathing, the exhaled air is warmer than the inhaled air which is at environmental temperature. The temperature difference between inspired and expired air can reach approximately 15 °C [87]. Therefore, different kinds of temperature sensors allow estimating $f_{R}$, based on the temperature difference between exhaled and inhaled air.

**Figure 4:**
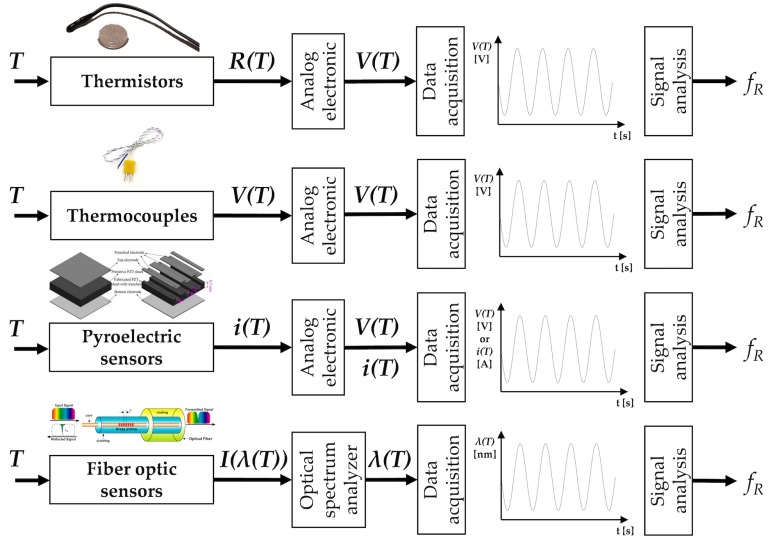
Temperature sensors: the main elements composing the measuring chain (sensor, analog electronics, data acquisition, post-processing or signal analysis stage) to estimate $f_{R}$. Analog electronic stages vary between different sensors since the output quantities are different. R(T), V(T), i(T), I(λ(T)) are resistance, voltage, current and light intensity changes caused by the temperature (*T*), respectively. V(T) is the voltage output. The pyroelectric sensor figure is adapted from [88]; the fiber-optic sensor figure is adapted from [89].

Different technologies and working principles can be used to sense temperature changes related to the breathing activity. From a plethora of available sensors, we report the most common electric-based sensors (i.e., thermistors, thermocouples, and pyroelectric sensors) that are largely employed for breathing patterns recording and for estimating $f_{R}$ values. In addition, a short description of FOSs is provided since these kinds of sensor are gaining interest in $f_{R}$ monitoring (see Figure 4).

### 4.1. Temperature Sensors

#### 4.1.1. Thermistors

The resistive thermometer (i.e., thermistor) is a type of resistor (made from certain metal oxides) for which resistance is dependent on temperature. There are two types of thermistors: (i) the negative temperature coefficient thermistors, which show a resistance drop when the surrounding temperature increases; (ii) the positive temperature coefficient thermistors which show an opposite behavior (the resistance increases with a temperature increment). Steinhart–Hart equation may express the nonlinear relationship between temperature (*T*) and the resistance (*R*) [47]. By using an approximation, the relationship can be expressed as in Equation (8):

(8) $$R=R_{0}\cdot exp\left[\beta \left(\frac{1}{T}-\frac{1}{T_{0}}\right)\right]$$

being *R* the resistance at any temperature *T*, $R_{0}$ the resistance at the reference temperature $T_{0}$, and β a constant that depends on thermistor material [47]. Due to the nature of the basic process, the number of conducting electrons increases exponentially with temperature. This pronounced non-linearity is a disadvantage of thermistors and limits their temperature span to about 100 °C. However, thermistors are characterized by high sensitivity in the temperature range of interest for respiratory monitoring (i.e., 10– 40 °C). Commercial thermistors are quite small (a few mm in diameter), but they have a quite high response time (typically some seconds) which can jeopardize a correct analysis of breathing pattern, especially in the FB case [90,91] and for real-time monitoring purposes. These sensors can be used to measure the respiratory signal at the level of the nose in adults and infants given their small size and low intrusiveness, in the clinical scenario. Since the sevenths, these sensors have been proven to be sufficiently accurate to detect regularity of $f_{R}$ and apnea stages when compared to gold standard techniques [92]. Given the relatively low cost (less than 10 dollars) and their ease-of-use, they are used as reference instruments in several studies as in [93,94], during QB.

#### 4.1.2. Thermocouples

A thermocouple produces a temperature-dependent voltage as a result of the thermoelectric effect (Seebeck effect), i.e., the occurrence of an electric potential occurs when two different metals are joined into a loop and the two junctions are held at different temperatures [95]. This Seebeck electromotive force (emf) is a voltage difference between the two ends of the conductor that depends on the temperature difference between the ends and a material property called the Seebeck coefficient $\sigma^{Seebeck}$ as in Equation (9):

(9) $$emf^{Seebeck}\left(T_{2}\right)-emf^{Seebeck}\left(T_{1}\right)=\int_{T_{1}}^{T_{2}}\sigma^{Seebeck}\left(T\right)dT$$

When wires of two different materials, *A* and *B*, are connected, the electromotive force that occurs depends on the temperatures of the free ends of the two wires and the temperature of the junction between the two wires. By considering the two free ends at temperature $T_{ref}$ and the junction at a temperature $T_{m}$, the voltage difference between the free hands may be written as in Equation (10):

(10) $$V=emf_{AB}^{Seebeck}\left(T_{m}\right)-emf_{AB}^{Seebeck}\left(T_{ref}\right)$$

being $emf_{AB}^{Seebeck}\left(T\right)=emf_{A}^{Seebeck}\left(T\right)-emf_{B}^{Seebeck}\left(T\right)$ the relative Seebeck emf of materials *A* and *B*. Some conductive materials and combinations are better than others to form a thermocouple [96]. The specific configuration may influence sensitivity, effects of corrosion, costs, and other characteristics. The accuracy is similar to that of thermistors, with typical values of 1 °C (or ± 0.75% of the measurement range) [47]. The response time of thermocouples is typically 0.5 s, although small-sized thermocouples allow obtaining shorter response time [47]. This property encourages the adoption of such sensors also in case of high values of $f_{R}$ which can occur in the clinical scenario [97]. Size of thermocouples which can be used for respiratory monitoring are comparable to those of thermistors, so their field of use is similar. Usually, the cost of thermocouples is higher than thermistors. Because of their size, these sensors may be integrated into face-masks or placed under the nostrils or into the nose for monitoring temperature during hypopnea [98,99,100,101].

#### 4.1.3. Pyroelectric Sensors

Sensors based on pyroelectric effect may also be used to monitor the temperature of respiratory airflow [91]. When a pyroelectric sensor is heated, the dipoles within the sensor exhibit random motion by thermal agitation. This phenomenon causes a reduction in the average polarization of the transducer, generating a charge build-up on the transducers. The output current (i.e., *i*) is proportional to the rate of temperature change. The output voltage produced by the transducer across the amplifier with an input resistance is related to the cross-sectional area of the transducer surface, the pyroelectric coefficient of the piezoelectric transducer, the upstream and downstream electrodes average temperature [102]. The *i* from the pyroelectric sensor can be calculated from the Stefan–Boltzmann law as in:

(11) $$i=\frac{2\cdot P\cdot \sigma_{B}\cdot a\cdot \gamma }{\pi \cdot H\cdot c}\cdot b\cdot T^{3}\cdot \frac{\Delta T}{L_{o}^{2}}$$

where $\Delta T=T_{b}-T_{a}$ is the temperature gradient between the object and its surroundings, *P* is the pyroelectric coefficient, $\sigma_{B}$ is the Stefan–Boltzmann constant, *a* is the lens area, γ is the lens transmission coefficient, *H* is the thickness, and *c* is the specific heat of the pyroelectric element, respectively, and $L_{o}$ is the distance to the object [103]. The *i* value is directly proportional to the ΔT.

Typical accuracy is ± 1 °C, while typical response time is comparable with that of thermocouples, or even better in the case of microfabricated pyroelectric sensors [104]. Since the sensors can assume different shapes as cylindrical [91] or thin films [105], they may be embedded in a face-mask [91] or on headphones near the nostrils [105] for respiratory monitoring purposes. In [105], an error of 1.12% on the average $f_{R}$ value was found compared to data recorded by a thermistor.

#### 4.1.4. Fiber-Optic Sensors

Recently, FOSs have been proposed to measure the temperature changes of the exhaled and inhaled air during breathing. Among others, FBG sensors have been used in the respiratory field. The principle of work is that the peak Bragg wavelength ($\lambda_{B}$) of an FBG sensor shifts to longer wavelengths as the temperature increases, and towards shorter wavelengths when the temperature decreases. The response is linear, and its temperature sensitivity coefficient is a function of the operating wavelength and the material’s properties of the fiber in which the FBG is inscribed [106]. This change may be expressed as follow [106]:

(12) $$\Delta \lambda_{B}=\lambda_{B}\cdot \left(\alpha +\frac{dn_{i}}{\frac{dT}{n_{i}}}\right)\cdot \Delta T=\lambda_{B}\cdot \left(\alpha +\zeta \right)\cdot \Delta T$$

being $n_{i}$ the refractive index of the fiber core, α the coefficient of expansion, ζ the thermo-optic coefficient of the fiber, and Δ*T* the temperature change. Peculiarities of these sensors are their small size (typical outer diameter of the fiber is 250 µm), the biocompatibility, the lightweight and the intrinsic safety because light powers them. For these reasons, sensors may be placed close to the patient nose or lips without causing discomfort and risks. Regarding the metrological properties, FBGs are characterized by a typical response time of 10 ms and sensitivity of 10.0 pm/°C that guarantee excellent performance in the monitoring of breathing even at high frequency [107].

Usually, these sensors are embedded into a small probe for monitoring breathing to avoid mechanical fiber breakage [108]. FBGs have been used to monitor the respiratory signal and $f_{R}$ by placing the sensor in the nasal-cavity during MR imaging procedure [109]. Alternatively, FBGs have been used to collect $f_{R}$ during mechanical ventilation by embedding the sensors in a small probe [108,109,110]. Despite one FBG is quite inexpensive and its cost is comparable to that of thermistors, the system used to interrogate the FBGs is expensive (more than 3000 dollars) and bulky. For this reason, this technology is mainly used for research purposes and tests in laboratory environment, or in particularly harsh environment (e.g., in the case of $f_{R}$ monitoring in MR room). In addition, FBGs are intrinsically sensitive to strain, so when used for temperature measurements it is pivotal to avoid or minimize mechanical strain.

Table 3 summarizes the main metrological properties and characteristics of the temperature sensors specific for $f_{R}$ monitoring, and their field of use.

### 4.2. Short Summary

Temperature sensors may be used for monitoring $f_{R}$. Depending on their response time (from 10 ms for FOSs up to some s for thermistors), temperature sensors can allow the monitoring of apnea, SB, QB, and FB. Thermistors typically present a quiet slow response time, hence their use is discouraged for quiet and fast $f_{R}$ monitoring, where other temperature sensors are preferable. Temperature sensors are usually placed at the level of the nostrils or/and lips and integrated into mechanical supports or face-masks to give mechanical stability to the sensor and reduce the sensitivity to body movements. All temperature sensors require to be exposed to the air exhaled and inhaled by the subject. For this reason, the measurements are intrinsically obtrusive; however, these sensors generally allow recording the respiratory waveform over time and the $f_{R}$ analysis on a breath-by-breath basis. In the case of FOSs, wires are needed to supply the power to the sensor and to deliver light. The sensitivity to environmental factors, the obtrusiveness and the presence of wires may limit the applicability of temperature sensors to indoor controlled environments (e.g., during imaging procedures) and in clinical settings (e.g., intensive care unit, geriatrics), while they are seldom employed in the monitoring of athletes especially in outdoor scenarios.

## 5. Techniques Based on Air Humidity

**Figure 5:**
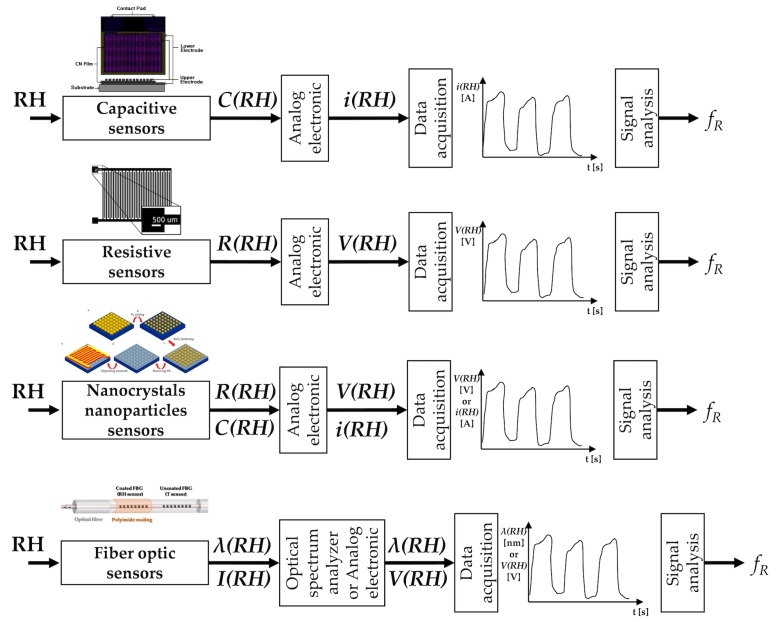
Relative humidity sensors: main elements composing the measuring chain (sensor, analog electronics, data acquisition, post-processing or signal analysis stage) to estimate $f_{R}$. Analog electronic stages vary between different sensors since the output quantities are different. C(RH), R(RH), λ(RH), I(RH) are capacitance, resistance, wavelength, and light intensity changes caused by the RH, respectively. V(RH) is the voltage output, i(RH) is the current output. The capacitive sensors picture is adapted from [112]; the resistive sensors picture is from [113]; the nanocrystals and nanoparticles sensors image is from [114]; the fiber-optic humidity sensors picture is adapted from [115].

The inhaled and exhaled air differ in the content of water vapor. Therefore, different types of sensors and configurations have been proposed to estimate the respiratory signal from the difference of water vapor contents between inhaled and exhaled air. Indeed, inhaled air is at environmental conditions (relative humidity (RH) from 40% to 80%), while the exhaled air is at body temperature and saturated by vapor (RH = 100%) [111]. Among others, electrical and FOSs have been mainly investigated for respiratory monitoring purposes (see Figure 5).

The principle of work of the electric humidity sensors is based on the sensitivity of one electric parameter to RH. The most common ones are based on the change of capacitance and of resistance with RH. Recently, new solutions based on nanoparticles, nanocrystals, and graphene have been investigated. Among FOSs, FBGs, and long-period grating sensors are used in this application.

### 5.1. Humidity Sensors

#### 5.1.1. Capacitive Sensors

The working principle of capacitive sensors for RH measurements is related to the capacitance change with the dielectric properties of the material interposed between the two electrodes of the capacitance [116]. This type of sensors is the most used in RH measurements, representing more than 75% of the market [117]. Although different configurations have been investigated, usually, a sandwiched structure with two electrode surfaces on each side or an interdigitated structure with comb electrodes are used. A dielectric material (i.e., polymer film or ceramic material) is deposited between the two electrodes; it is usually made of a porous material that either absorbs water vapor when the environmental RH rises or releases it when RH decreases. The dielectric properties of the material change with the amount of absorbed water vapor and therefore with the environmental RH. Since the sensor’s capacitance depends on the dielectric properties, its changes can be used as an indirect measurement of RH. Usually, an appropriate electronic circuit allows transducing the capacitance within an electric signal that represents the sensor output. The estimation of the $f_{R}$ is based on the cyclic changes of the sensor’s output due to RH changes during the two respiratory phases. These sensors show good linearity and accuracy (around 3%); the rise time is usually of some seconds although faster solutions have been proposed [116].

#### 5.1.2. Resistive Sensors

The principle of work of resistive sensors for RH estimation is based on the electrical impedance change with humidity. Therefore, RH values are estimated starting from the changes of electrical conductivity of the sensing films [116]. A specific electronic circuit allows the transduction of the resistance into a voltage signal, which represents the sensor’s output. Several studies have shown the potential of resistive RH sensors for monitoring $f_{R}$ and for detecting apnea stages. During the 1980s, this capability was shown on patients and volunteers using a resistive sensor with a rise time of almost 10 s [118]. Faster resistive sensors have been proposed in the literature and assessed in the laboratory or using simulated breathing patterns [119,120,121,122]. Recently, a new instrumented mask embedding one of this type of sensor has been assessed on humans, showing good performance in the estimation of $f_{R}$ (limits of agreement of ±1 bpm) [10,123]. Resistive RH sensors have also shown good performance up to very high $f_{R}$ values (e.g., up to 240 bpm) [10]. Recently a fast and ultra-sensitive electrical humidity sensor was also tested for $f_{R}$ monitoring on humans and on rats [124]. Thanks to the high resolution (0.1%) and fast response time (37 ms) it was able to monitor the $f_{R}$ on rats that have smaller tidal volume and higher $f_{R}$ than humans.

#### 5.1.3. Nanocrystals and Nanoparticles Sensors

The investigation of sensors for RH monitoring fabricated with nanocrystals, nanoparticles and graphene-based nanocomposites has been encouraged by the high ratio between exchange surface and volume, so different solutions have been proposed [125,126,127]. For instance, the resistance of a thin film of SiO_2_ nanoparticles which non-linearly changes with RH has been proposed for $f_{R}$ monitoring [125]. The response and recovery time of the sensor were approximately 1 s. The authors assessed the capability of the sensor to follow $f_{R}$ values up to approximately 100 bpm at rest and it is also able to both monitor respiratory signal and discriminate respiratory phases during exercise. The same group fabricated a flexible nanocrystal-based RH sensor with a response/recovery time of 40 ms [128]. The system was assessed on humans and showed the capability to follow breathing patterns, although its output was not compared with a reference system. A cost-effective sensor ($0.005 for materials for the paper chip and $1.50 for the mask) based on the changes of resistance of a paper with printed graphite electrodes has been also investigated for respiratory monitoring [129]. The sensor was embedded into a mask and showed the ability to follow $f_{R}$ at rest and during walking up to 60 bpm.

#### 5.1.4. Fiber-Optic Sensors

RH sensors based on fiber optics are gaining broad acceptance for application in $f_{R}$ monitoring. Among others, FBGs and long-period grating sensors have been largely investigated in this field [130,131]. Since they can sense only physical parameters (i.e., temperature and strain), polymer and nanoparticle-based coatings are used to make FBGs and long-period gratings sensitive to RH [132,133]. The coatings are made of hygroscopic materials that absorb or desorb environmental water vapor in response to RH changes. This phenomenon induces changes in the gratings behavior making it able to detect RH changes [134,135,136]. Also, interferometric-based FOSs have been used for this application. Solutions based on hygroscopic materials which cause a shift in the interference pattern due to the sorption of water vapor have been proposed [137].

The metrological properties of these sensors strongly depend on the material coatings and the coating thickness. For instance, the response time ranges from tens of ms to tens of s [138,139]. The capability of RH sensors based on fiber-optic technology to monitor $f_{R}$ has been investigated using several configurations. Interferometric-based ones have shown the ability to follow breathing patterns [140], but the authors did not used a reference to assess the performance of the sensor. Sensors based on optical fiber gratings have been tested during mechanical ventilation [110,141,142]. The sensor was assessed by analyzing its capability of estimating the $f_{R}$ during mechanical ventilation from 12 bpm to 24 bpm [143] and on humans, during QB. During the assessment on humans the results provided by the proposed system were compared with $f_{R}$ values estimated by a reference (i.e., by a spirometer). The experiments showed good agreement between the two techniques with an absolute value of the percentage errors lower than 2.07%, with $f_{R}$ values up to 35 bpm [144]. A simple intensity-based sensor able to discriminate inspiratory and expiratory phases due to the condensation on an optical fiber tip placed in front of one of the two nostrils has been also assessed on 10 smoking men. The system showed the ability to follow the breathing signal during QB and to discriminate apnea phases [145].

Table 4 summarizes the main metrological properties and characteristics of the RH sensors specific for $f_{R}$ monitoring, and their field of use.

### 5.2. Short Summary

RH sensors allow monitoring $f_{R}$ by detecting the difference of water vapor content between inspiratory and expiratory air. Therefore, they need to be exposed to the inhaled and exhaled air, with consequent invasiveness, slightly mitigated by the small size of some solutions. Furthermore, RH is usually detected at the level of the nostrils or/and mouth, sometimes by using sampling face-masks. Electric sensors, especially capacitive ones, are the most commonly used for $f_{R}$ estimation, but solutions based on fiber-optic technology and nanoparticles are starting to attract the interest of scientists. Given the good metrological characteristics, all RH sensors can be used to record the respiratory waveform over time. These sensors have been used in extremely structured environments (e.g., intensive care unit), but rarely during exercise and in outdoor applications.

## 6. Techniques Based on Air Components

During breathing, the content of oxygen and carbon dioxide (CO_2_) differs between the inhaled and exhaled air as a result of cellular respiration. The CO_2_ percentage fluctuates from typically 0.04% (<300 parts per million, ppm) during inhalation up to 6% (around 60,000 ppm) during exhalation [146]. Therefore, CO_2_ variations can be used to estimate $f_{R}$.

**Figure 6:**
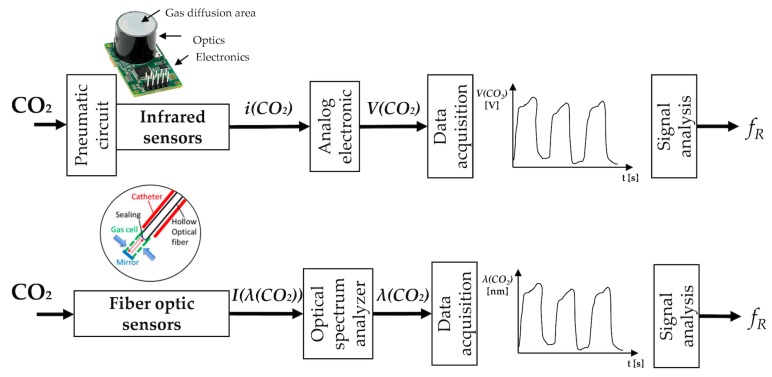
CO_2_ sensors: the main elements composing the measuring chain (sensor, analog electronics, data acquisition, post-processing or signal analysis stage) to estimate $f_{R}$. Analog electronic stages vary between different sensors since the output quantities are different. $i(CO_{2})$, $I(\lambda \left(CO_{2}\right))$ are current and light intensity changes caused by the CO_2_, respectively. $V(CO_{2})$ is the voltage output. The scheme of fiber-optic sensors is adapted from [148].

Different kinds of chemical sensors are used for the estimation of $f_{R}$ starting from the CO_2_ concentration difference between exhaled and inhaled air. Among others, electrical and optical sensors are the most used at this scope (see Figure 6). A technique that uses sensors for measuring human respiration CO_2_ from exhaled gases is known as capnography. The typical pattern of a capnogram (i.e., expiration, alveolar plateau, and inspiration phases) can be found in [147].

### 6.1. CO_2_ Sensors

#### 6.1.1. Infrared Sensors

Infrared sensors are used to detect CO_2_ in a gaseous environment by its characteristic absorption, working as spectroscopic sensors. The estimation of $f_{R}$ with such kind of sensors mainly requires the use of a nasal cannula or a mask to convey the inhaled and exhaled gas to the sensor. The key components of the measuring chain are an infrared source, a light tube, an interference (wavelength) filter, and an infrared sensor. The gas is pumped or diffuses into the light tube, and the absorption of the characteristic wavelength of light is measured. Among others, nondispersive infrared sensors (NDIR) are the most often used for measuring CO_2_. These sensors can have a discrimination threshold of 20–50 ppm. Their accuracy is usually of ±0.3% CO_2_ (±300 ppm). The principle of work of NDIR gas sensors is based on the Beer-Lambert Law [149]:

(13) $$I=I_{0}\cdot exp\left(-k_{abs}\cdot C_{gas}\cdot L_{opt}\right)$$

being $I_{0}$ the initial radiation beam intensity, *I* is the beam intensity after traversing the gas to the detector, $k_{abs}$ is an absorption coefficient, $C_{gas}$ is a gas concentration, $L_{opt}$ is the sample optical path length defined typically by the effective sample chamber length of the sensor [149].

Shorter response time values (up to <100 ms) are typical of NDIR gas sensors which use the flow through sample method, compared to diffusion sampling method (>10 s). To implement the flow through sample method, the following stages upstream the sensor are required: the gas output sampling with a tube, a particulate filter, a water trap (or Nafion tube that contains a highly selective, semi-permeable membrane to water vapor), a hydrophobic filter, a pump (which eventually may be placed downstream of the sensor). All these elements required for the measurement let the price rise (typically 300–1000 dollars). It is also crucial to highlight that such kinds of sensors require a calibration (The calibration procedure is strictly required for the accurate measurement of the CO_2_ concentration. Given that the CO_2_ level strongly varies during breathing activity, the outputs of CO_2_ sensors may potentially be used to accurately estimate $f_{R}$ even without a recurring calibration.) each time they are used for collecting CO_2_ and a warm-up time (i.e., the time elapsed from sensor power to the time when the sensor output value is stable) is usually 60–120 s.

CO_2_ sensor may be positioned inside the main unit, away from the subject or between the endotracheal tube and the breathing circuit, especially in clinical settings. Sensors may be placed at the end of the mask of the nasal cannula to perform partial or total sampling of CO_2_ [150]. A recent patent of Medtronic introduced a new approach for the CO_2_ sampling, which can be used to sample CO_2_ in the case of concurrent delivering oxygen to one nostril and when one or both nostrils are blocked (e.g., deviated septum, sinus congestion). This approach is based on CO_2_ collection at the level of the lips [151]. Yang et al. [152] have positively tested the ability to estimate $f_{R}$ in real time with a low-power and portable device. The system was used in conjunction with modulation technology to eliminate the fluctuations at the trough of the waveform of the CO_2_ concentration signal.

#### 6.1.2. Fiber-Optic Sensors

FOSs have been extensively used as sensors for gas concentration. Generally, the fiber is used to transfer the light and to investigate variation at the fiber’s distal end where a CO_2_ sensitive material is positioned. Considering the spectrum of absorption of the CO_2_, two main kinds of light can be used: near-infrared light (range of wavelengths 0.7–1.4 µm) and mid-infrared light (range of wavelength 1.6–5.0 µm). Long-period fiber gratings [153,154] and optical fiber-based Fabry Perot interferometer [155] have been used with near-infrared light. Additionally, cheaper systems based on mid-infrared light (with a wavelength of around 4.2 µm that is strongly absorbed by CO_2_) to directly detect molecular absorption of CO_2_ have also been proposed. Flexible hollow optical fiber that shows low transmission loss for mid-infrared light has been used. Recently, it has been developed a small gas cell attached to the distal end of a thin optical fiber probe that could be inserted into airways via a catheter or a bronchoscope [148]. The target gas penetrates the cell through small holes. A polyvinylidene chloride film is attached to the distal end of the hollow optical fiber. The measurement of the CO_2_ is then obtained with Fourier-transform infrared spectroscopy. Measurement error of the mentioned system is ±0.3%, minimum threshold of 0.45% of CO_2_ [148].

In Table 5 the main metrological properties and characteristics of the air component sensors specific for $f_{R}$ monitoring—and their field of use—are reported.

### 6.2. Short Summary

CO_2_ sensors may be used to monitor $f_{R}$, given their good accuracy and response time. Infrared sensors are the most common sensors both in clinical settings and during sporting activities in indoor structured environments. All the sensors presented for respiratory monitoring purposes require to be exposed to the air exhaled by the subject, so the measuring systems are intrinsically quite intrusive, requiring pneumatic connections and tubes to sample the gas. FOSs are attracting interest in real-time $f_{R}$ monitoring starting from the analysis of air concentration; however, only a few examples are reported in the literature with explicit tests on humans during breathing. Commonly, CO_2_ is sampled at the level of the nostrils or lips and usually sampling face-masks are used. This kind of measurement is quite insensitive to body movements. One of the main drawbacks of CO_2_ sensors is their cross-sensitivity to other gas components and to environmental factors changes (i.e., humidity, temperature). Furthermore, both electrical and optical sensors need numerous stages to process the measurand and to extract the breathing pattern, hence these techniques are mainly used in clinical settings (e.g., intensive care unit), while they are seldom employed in the monitoring of athletes especially in outdoor scenarios. Nevertheless, CO_2_ sensors are suitable for the continuous recording of the respiratory waveform over time.

## 7. Techniques Based on Chest Wall Movement Analysis

Breathing is made possible by the work of the respiratory muscles, with the diaphragm and external intercostal muscles playing a major role during inspiration. The diaphragm contracts itself and moves downward producing a pressure difference causing air to enter the lungs. The contraction of the intercostal muscles causes the ribs to elevate which results in the expansion of the chest cavity allowing a greater volume of air to enter. This amount of air within the lungs causes an expansion of the chest wall diameter up to ∼7 cm [156]. For this reason, techniques based on the analysis of chest wall movement have been extensively explored. Among others, three approaches have been mainly investigated: (i) based on the record of chest wall strain caused by the respiratory activity, (ii) based on the transthoracic impedance change and (iii) based on the record of thorax tridimensional movements (e.g., thorax inclination, acceleration, and velocities).

### 7.1. Strain Sensors

**Figure 7:**
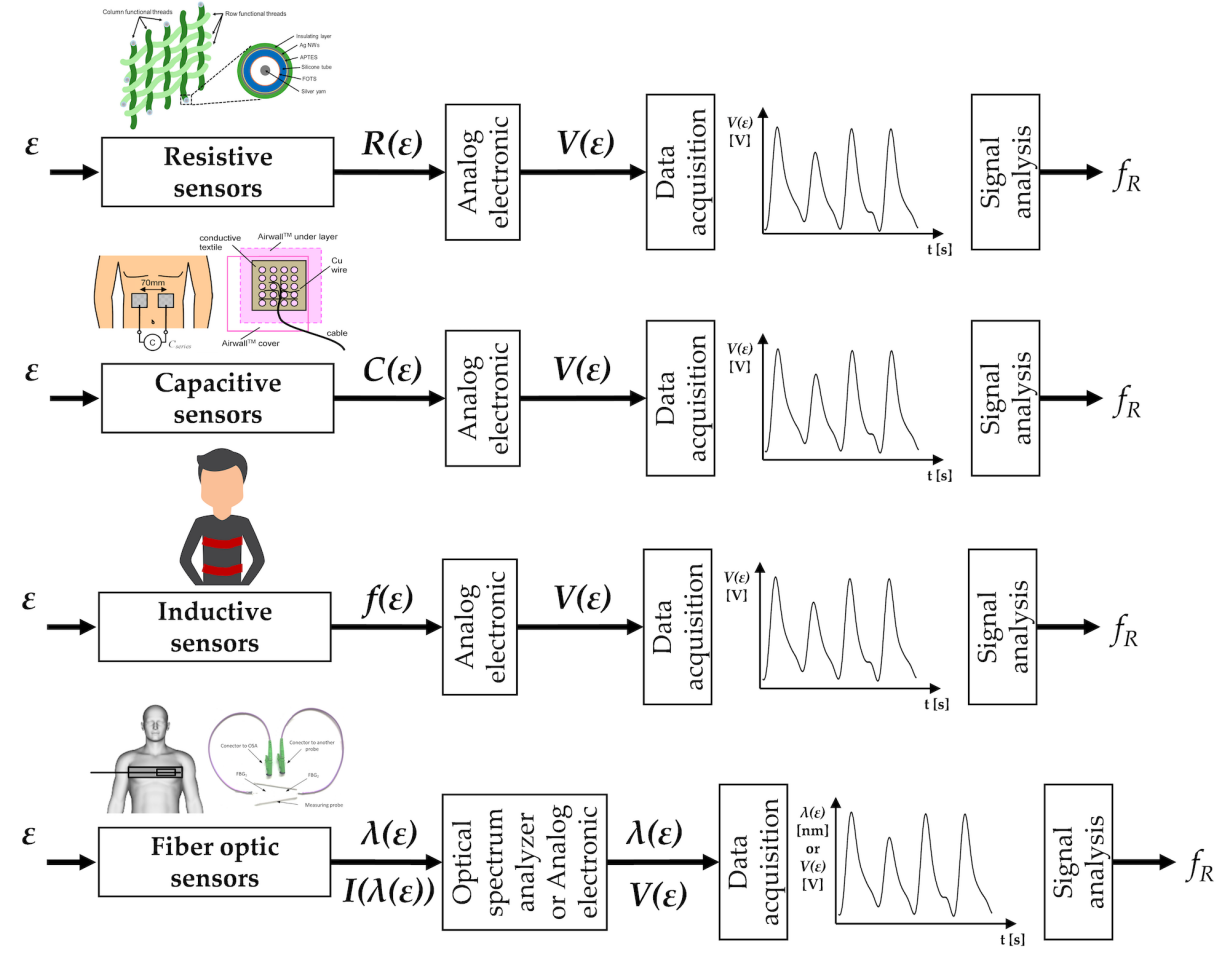
Strain sensors: the main elements composing the measuring chain (sensor, analog electronics, data acquisition, post-processing or signal analysis stage) to estimate $f_{R}$. Analog electronic stages vary between different sensors since the output quantities are different. R(ϵ), C(ϵ), f(ϵ), λ(ϵ), I(λ(ϵ)) are resistance, capacitance, frequency peak, wavelength and light intensity changes caused by the strain (ϵ), respectively. V(ϵ) is the voltage output. The resistive sensors picture is from [158]; the capacitive sensor picture is adapted from [159]; the fiber-optic sensors picture is adapted from [160].

Several methods exploit the deformations of the chest wall for the indirect measurement of respiratory activity. Strain sensors are used to transduce the cyclic expansion and contraction of the chest cavity in a signal that can be managed to extract breathing pattern and $f_{R}$ values. The working principle and output signals of the strain sensors are reported in Figure 7.

#### 7.1.1. Resistive Sensors

The working principle of the piezoresistive strain sensors is based on the variation of electrical charge concentration in response to externally applied strain, which modifies the geometrical shape of the piezoresistive sensing element. These changes in shape can be measured, and the strain can be determined. In piezoresistive strain gauges, the strain is related to the change in resistance. The performance of these sensors is generally reported by referencing the gauge factor ($k_{G}$) according to the following formula:

(14) $$\frac{\Delta R}{R_{0}}=k_{G}\cdot \frac{\Delta L}{L_{0}}$$

where $R_{0}$ is the resistance in the absence of strain, $L_{0}$ is the length in the absence of strain, ΔR and ΔL are the changes in resistance and length due to an applied mechanical strain. Details on the theory and technology of piezoresistive sensors can be found in [157]. Over the mechanical strain limit of the sensor, the output may become unstable due to excessive strain of the sensing element and mechanical failure of the sensing element may occur.

Two main approaches can be used for manufacturing piezoresistive textile-based strain sensors: (i) fabrics are coated with conductive polymers or elastomers [161] and (ii) conductive yarns are embedded into the textile structure during the manufacturing process [162,163].

When piezoresistive fabrics or conductive yarns embedded into textile are specifically intended for the use as breathing-related strain sensors, the elasticity of the fabrics and textiles is an essential property. There are two main limits of this type of sensors: (i) issues related to their durability, so possible shortcomings are poor repeatability and performance deterioration after washing or repeated folding [164], and (ii) the sensor output may be affected by motion artifacts (i.e., breathing-unrelated movements, such as walking and speaking). Therefore, specific filters in the post-processing phase have to be used [165,166]. Generally, performance improves when the sensor is positioned at the level of the upper thorax that is less susceptible to body motion artifacts during walking and running in comparison to the abdominal area [165].

Several measuring systems for respiratory activity monitoring based on the integration of one or more strain sensors have been designed. Two sensors have been used in [167] to record both the thoracic and abdominal strain. The obtained measuring system can be used to detect the $f_{R}$ during QB [167]. A weft-knitted strain sensor has been proposed in [165] and successfully used to detect apnea stages as well as SB, QB, and FB. Piezoresistive sensors have been integrated into automobile safety belt to monitor respiration in [168]. Although there are many studies that use these sensors in the respiratory field, only a few of these have carried out a quantitative comparison against reference values gathered by gold standard instruments. When available, generally the comparison takes into account average $f_{R}$ values calculated in fixed time windows [169]. Good performance was reported in [170], where the use of one piezoresistive sensor allowed estimating $f_{R}$ with a maximum error of 1.8 bpm when compared to reference values (i.e., by spirometer) during QB. Rarely, studies have compared the performance of piezoresistive sensors on a breath-by-breath basis against a reference instrument [170]. Average values of $f_{R}$ have been recorded with piezoresistive sensors during exercise (walking, running, and cycling) [166]. The worst results were obtained during running activity, with a bias of ∼1.01 ± 4.01 bpm when compared against reference values (i.e., by a spirometer) [166].

#### 7.1.2. Capacitive Sensors

The alternation of inhalation and exhalation determines changes in capacitance that can be registered at the abdomen or chest levels.

The human body is mainly composed of water (permittivity ϵ = 80) and a conductive electrolytic solution, while the air that flows in and out of the body during breathing is nonconductive and has a lower permittivity (ϵ = 1) than water [159]. Considering a configuration where an electrode is placed on the anterior part of either the chest or the abdomen and another electrode is placed on the corresponding posterior side, both permittivity and between-electrode distance change during breathing. Consequently, capacitance decreases during inhalation and increases during exhalation. By forming a capacitor area between the two electrodes, inhalation and exhalation can be detected by measuring the capacitance, *C*, according to the following equation:

(15) $$C=\epsilon_{R}\cdot \epsilon_{0}\cdot \frac{S_{e}}{d_{e}}$$

where $S_{e}$ is the electrode area, $d_{e}$ is the distance between the electrodes, $\epsilon_{0}$ the vacuum permittivity and $\epsilon_{R}$ the relative permittivity of the inner body material placed between the electrodes [159].

The sensitivity of sensors relies on electrodes number, size, the distance between them and the position of the electrodes [171]. Electrodes could be flexibly or tightly connected. When electrodes are embedded into a flexible, elastic connecting belt, the measuring device can be better adjusted to the human body; tight electrodes eliminate *C* changes caused by electrodes movements and significantly reduce noises in output signals [172].

The sensors’ outputs allow for the identification of the respiration cycle and the distinction between different breathing modes (i.e., quiet, panting, apnea, and deep breathing) [171]. Capacitance meter can be used to record *C* changes [171,173], while sophisticated circuit may promote the use of such kind of sensors to develop smart garment [172,174,175,176]. During QB respiration (on two healthy subjects, 50 min), a comparison with a reference instrument demonstrated excellent performance in the $f_{R}$ monitoring with a bias of 0.01 ± 1.90 breaths/min [177]. When tested on 9 patients with COPD at rest (after exercise), capacitive sensors demonstrated excellent performance when monitoring $f_{R}$ values over 2 min (bias of −0.14 ± 0.55 bpm) [177].

#### 7.1.3. Inductive Sensors

Direct current-coupled inductive sensors can be used to register changes in the self-inductance and oscillatory frequency of coils encircling the rib cage and abdomen. This method can be used to monitor the volume and time components of the breathing pattern [178]. Generally, the monitoring device consists of one or two elastic cloth bands, which contain insulated wires encircling the rib cage and/or the abdomen. These wires are connected to an oscillator module that oscillates at a low-amplitude wave. Respiratory activity produces variations in the self-inductance of the coil, thereby changing frequency. This frequency is then demodulated to provide an analog signal related to the rib cage and abdomen strains.

Each coil forms the inductance element of a resonant circuit maintained in oscillation at a frequency chosen to avoid interference with other medical equipment:

(16) $$f=\frac{1}{\pi }\sqrt{\frac{1}{L_{i}\cdot C}}$$

where *f* is the oscillation frequency, $L_{i}$ the inductance and *C* is related to circuit capacitance [179].

This method is one of the most widespread in strain-based respiratory monitoring, and it has been widely adopted in several commercial products, including LifeShirt (VivoMetrics Inc., Ventura, CA, USA). Several studies have tested measuring systems based on inductive sensors on healthy subjects (in the standing, supine, sitting, prone and semi-recumbent postures [178]), on critically ill patients [180], in newborns [44], and in children [181]. Wakefulness and sleep in patients with obstructive sleep apnea have also been investigated with two inductive sensors embedded at the level of upper thorax and abdomen [182]. They have also been used to investigate the breath-by-breath variability from thoraco-abdominal strain signals collected in healthy subjects [183]. Literature reports robust studies aiming at comparing the performance of systems based on inductive sensors and reference systems in a wide range of scenarios and $f_{R}$ values. Systems have been tested during an incremental running test on a treadmill, and multiple periods of recovery [10], and during cycling [184]. On patients with respiratory issues, similar thoraco-abdominal asynchronies and $f_{R}$ values were found when comparing a two-coil inductive sensor with a motion capture system (requiring complex model and markers to be attached on the chest [185]) both during quiet breathing and exercise [186]. Good performance have also been reported when testing a smart t-shirt instrumented by inductive sensors on healthy subjects [187]. Specifically, a bias of 0.01 ± 0.11 s (mean error 2%) was found during walking and running across different exercise–intensity domains, when analyzing breath-by-breath values [187]. Similar results were obtained on heart failure and COPD patients, with the sensors placed around the rib cage and abdomen at the level of the nipples, and of the umbilicus [187].

#### 7.1.4. Fiber-Optic Sensors

The use of FOSs is gaining large acceptance as an alternative to electrical and mechanical sensors for monitoring strains. FOSs can be designed using different working principles. FBGs and intensity-based FOSs are the most used technologies to develop systems for respiratory monitoring. An extensive review on this topic has been recently published [188], while health supervisions applications through the use of FOSs embedded in textile have been reviewed in [189]. FBGs typically have short response time (approximately 10 ms) and high strain sensitivity (typically 1 nm/mϵ). These features allow monitoring both rapid and small chest wall movements.

Numerous papers have investigated the use of patches and or instrumented mattresses or cushions embedding one or more FBGs. Such solutions exhibited errors always lower than 12% when compared to reference values [190], or bias of ±1 bpm in [191]. The integration of FBGs in wearable mechanical structures used to amplify the heartbeat and the sensitivity to respiration has also been proposed, as in [192] where a single FBG has been tightly bonded across the diaphragm using a thin layer of cyanoacrylate adhesive. Smart textiles based on more FBGs have been developed and tested. Solutions based on 2 sensors [193], 6 sensors [194], or 12 sensors [46,195,196] have been proposed. The system embedding 12 sensors showed a bias of −0.02 ± 2.03 bpm against a reference system (i.e., a marker-based motion capture system) when breath-by-breath $f_{R}$ values were estimated on healthy volunteers during QB [195]. Different solutions based on signal intensity changes of optical fibers integrated into textile have also been proposed to measure average $f_{R}$ [197,198,199]. In supine position during MR scan, a bias of 0.01 ± 2 bpm has been found comparing data retrieved by a fiber-optic-based garment with data recorded by an MR-compatible bellow (used as reference instrument) [200].

Table 6 summarizes the main metrological properties and characteristics of the strain sensors specific for $f_{R}$ monitoring, and their field of use.

### 7.2. Impedance Sensors

#### Transthoracic Impedance Sensors

**Figure 8:**
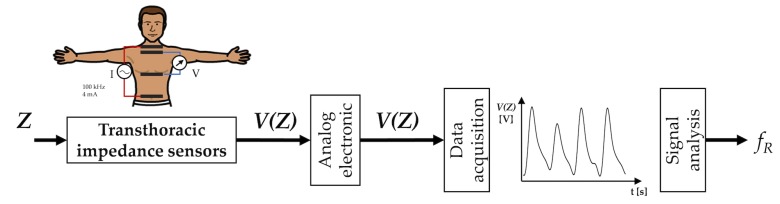
Impedance sensors: the main elements composing the measuring chain (sensor, analog electronics, data acquisition, post-processing or signal analysis stage) to estimate $f_{R}$. V(Z) is the voltage output caused by impedance (*Z*) changes.

Cyclic change of thoracic impedance occurs during breathing. Changes in the electrical resistance of the lungs are mainly a result of the following two effects: (i) during inspiration, there is an increase in the gas volume of the chest in relation to the fluid volume, which decreases conductivity; (ii) during inspiration, the length of the conductance paths increases because of expansion. Both of these effects cause an increment of the electrical impedance during inspiration. The relationship between this impedance change and the respiratory volume is approximately linear. Therefore, transthoracic impedance sensors provide a way to measure respiratory activity by sensing these changes (see Figure 8).

This method is based on the use of electrodes (either two or four) placed on the chest of the subject. The electrocardiogram electrodes can be used for respiration as well by taking advantage of this measuring technique [201,202]. Considering a four-electrode configuration, a high-frequency (typically ∼50 kHz) and low-amplitude current (less than 1 mA) is injected by two electrodes on the thorax, whereas the other two electrodes are used to record the *Z* changes by measuring the voltage changes between them. When measuring respiratory activity, the thorax presents an electrical impedance to the electrode that consists of two impedance components: an almost constant value and a varying value. The relatively constant value is the baseline impedance (typically 500 Ω). The varying value is known as respiratory impedance.

The *Z* change, ΔZ (expressed as Ω/m^3^), can be described with the following equation:

(17) $$\Delta Z=\int_{v}\;\frac{1}{\Delta \sigma }\cdot J_{LE}\left(t_{0}\right)\cdot J_{LI}\left(t_{1}\right)\cdot dv$$

where $t_{0}$ and $t_{1}$ are two time instants, Δσ the conductivity change between the two time instants, $J_{LE}$ the lead field of the voltage measurement electrodes for unit reciprocal current, $J_{LI}$ the lead field of the current feeding electrodes for unit current, and *v* the conductor volume [203]. Even though the two-terminal measurement configuration is the most used, it introduces some measuring errors. They are mainly related to nonlinear voltages (recorded by the two electrodes) generated by the current flowing through the polarization impedance at the electrode-tissue interface and overlapped to the signal of interest [202]. The four-electrode configuration yields a more accurate measurement because the sites of current injection and voltage measurement are physically separated, but require two additional electrodes [201].

This technique has shown promising results for long-term monitoring of respiration during sleep in adult males [204,205]. Performance in terms of $f_{R}$ estimation has been tested in different postures (i.e., supine, left-lateral lying, right-lateral lying, sitting, standing) and during different activities (i.e., slow walking, fast walking, slow running, fast running) in [206]. Correlation coefficients were always higher than 0.75, with excellent results even during running and walking (coefficients always > 0.93) [206]. The posture specificity of the method has also been investigated in [207]. By analyzing the power spectrum of the respiratory impedance curve, average values of $f_{R}$ of very preterm infants have been investigated in [208]. Impedance sensors have also been used to identify childhood pneumonia [209]. Besides, a pacemaker integrated with transthoracic impedance sensor has been developed and tested for sleep apnea screening [210]. Recently, the use of advanced data analysis techniques (i.e., empirical mode decomposition—EMD) has been tested to improve the accuracy in capturing respiratory episodes from thoracic impedance raw signals [206]. As in the case of other strain-based methods, the transthoracic impedance measurement is affected by movement artifacts. Simultaneous use of a piezoresistive-based t-shirt and a transthoracic measurement system during four physical exercises in a gym (i.e., walking, running, cycling, elliptic exercises) have been tested in [166]: piezoelectric sensors showed better performance in terms of robustness and reduced susceptibility to movement artifacts.

Table 7 summarizes the main metrological properties and characteristics of the impedance sensors specific for $f_{R}$ monitoring, and their field of use.

### 7.3. Movement Sensors

**Figure 9:**
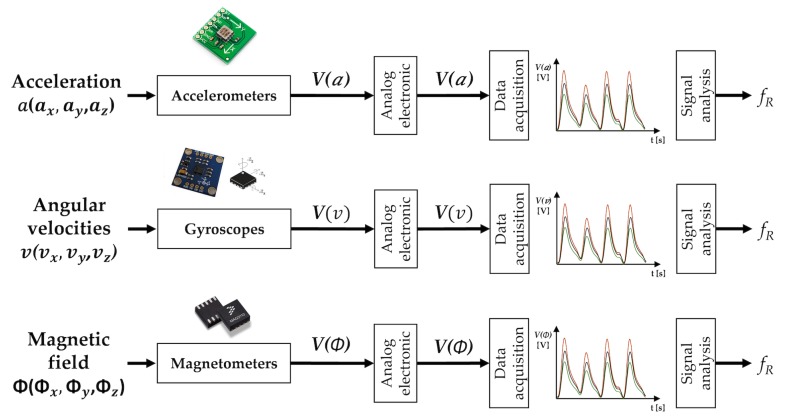
Movement sensors: the main elements composing the measuring chain (sensor, analog electronics, data acquisition, post-processing or signal analysis stage) to estimate $f_{R}$. The output quantities are different among sensors. $\left(a_{x},a_{y},a_{z}\right)$, $\left(v_{x},v_{y},v_{z}\right)$, and $\left(\Phi_{x},\Phi_{y},\Phi_{z}\right)$ are three-axis accelerations, three-axis angular velocities, and three-axis magnetic field outputs changes caused by the acceleration (*a*), angular velocity (*v*) and magnetic field (Φ) changes, respectively. V(.) is the voltage output.

Different sensors may be used to record data related to chest movements. Acceleration forces, angular velocities variations and changes in the magnetic field allows recording breathing-related data. Generally, movement sensors are placed on the chest wall of the subject so that they can follow its movement. Sensors working principles and output signals are summarized in Figure 9.

#### 7.3.1. Accelerations Sensors (Accelerometers)

An accelerometer is an electromechanical device that converts mechanical motion into an electrical signal. It measures acceleration forces caused by either gravity or motion. Different transduction elements can be used to achieve this goal. Basically, as the small accelerometer mass reacts to motion, it places the transduction element (i.e., capacitive, piezoelectric, piezoresistive sensor) into compression or tension. As the human body moves, the mass of the accelerometer, *m*, moves with an inertial response. The stiffness of the spring, $k_{el}$, provides a restoring force to move *m* back to equilibrium while internal frictional damping, *c*, opposes any displacement away from equilibrium. Independently from the working principle, a model of this measuring device may be exemplified with the following equation:

(18) $$m\cdot \frac{d^{2}y}{dt^{2}}+c\cdot \frac{dy}{dt}+k_{el}\cdot y=c\cdot \frac{dx}{dt}+k_{el}\cdot x$$

where *y* denotes the position of *m* within the accelerometer and *x* is the displacement of the human body. The wide bandwidth (hundreds of Hz) allows real-time monitoring in SB, QB, and FB [211].

To date, accelerometers-based methods for measuring breathing-related movements have been roughly validated, and rarely research has addressed the problems caused by breathing-unrelated movements as in [212]. Breathing pattern can be recorded with one or more accelerometers at the level of the upper thorax and/or abdomen [213,214]. When single- or dual-axis accelerometers are used, attention must be paid to appropriately align sensors with the major axis of rotation to improve the measurement accuracy. Conversely, the use of a triaxial device allows measuring acceleration regardless of body orientation [212,215]. A triaxial accelerometer positioned at the level of lower costal margin allows the accurate measurement of $f_{R}$ on seated subjects: $f_{R}$ values compared to those recorded by a reference instrument (i.e., pressure at the level of the nose) showed a correlation coefficient of 0.98 and a root mean square error of 0.38 bpm (maximum error ∼3 bpm) [212]. Average $f_{R}$ values estimated by the accelerometer signal were correlated with those from a reference instrument (i.e., spirometer), with an error <1% during QB [216]. Furthermore, different breathing patterns have been monitored with one or more accelerometers (e.g., quiet, bradypnea, tachypnea, Cheyn-stokes, Kaussmal, Biot’s) in [216] and $f_{R}$ values were extracted. When accelerometers were used during walking (2.5 km/h) and running (6 km/h) activities, the error increases up to 7.45 bpm and 4.52 bpm, respectively, when the information of three axes were merged and used as reported in [217]. Similar results were found during walking and running (mean percentage error of 8.59% and 4.13% during walking at 5 km/h and running at 15 km/h, respectively) in [218].

#### 7.3.2. Angular Velocities Sensors (Gyroscopes)

A gyroscope is a sensor mounted on a frame and able to record an angular velocity if the frame is rotating. Many classes of gyroscopes exist, depending on the operating physical principle and the involved technology. Mechanical and micro-electromechanical system (MEMS) gyroscopes are the most commonly used. MEMS gyroscopes are movement sensors able to detect and measure the rate of rotation of an object around a particular axis (i.e., 1-axis, 2-axis, and 3-axis).

The working principle of a gyroscope is widely described in [219]. Angular velocities ($p_{x}$, $p_{y}$, $p_{z}$) and Euler angles (ω, θ, ϕ) are related as follows:

(19) $$\left[\begin{array}{c} \omega^{\prime }\\ \theta^{\prime }\\ \phi^{\prime } \end{array}\right]=\left[\begin{array}{ccc} 1 & \sin\phi \cdot \tan\omega & \cos\phi \cdot \tan\omega \\ 0 & \cos\phi & -\sin\phi \\ 0 & \sin\phi \cdot \cos\omega & \cos\phi \cdot \cos\omega \end{array}\right]\left[\begin{array}{c} p_{x}\\ q_{y}\\ r_{z} \end{array}\right]$$

where $p_{x}$ is the angular velocity generated by rotation of the x axis, $q_{y}$ is the angular velocity generated by rotation of y axis, $r_{z}$ is the angular velocity generated by rotation of the z axis [220]. According to Equation (19), estimation of the change in the thorax angle using only a gyroscope has to entail integral calculation. In this process, the sensor drift may cause a divergent output determined by the integration of not only the change in the thorax angle but also the errors of the gyroscope sensor [221]. For this reason, gyroscopes are usually used in conjunction with an accelerometer. When a triaxial accelerometer is employed, the 3-axial angular velocities are merged with 3-axial accelerations [222]. Therefore, gyroscopes allow producing high-quality respiration signals to overcome the weakness of using a single accelerometer [216], even during exercise [220].

Several metrological characteristics (detailed in [223]) have to be considered when a gyroscope is intended to be used to estimate $f_{R}$. The wide bandwidth (more than 100 Hz) allows real-time monitoring in SB, QB, and FB [224].

When a gyroscope was used in conjunction with a triaxial accelerometer on healthy subjects in stationary conditions, the average values of $f_{R}$ showed discrepancies always lower than 1.5 bpm (average error of 0.77 bpm), when compared to values manually counted by the authors [222]. Respiratory patterns obtained fusing accelerometer and gyroscope signals were investigated in [225]: the average error in estimating average $f_{R}$ values was 0.7 bpm during QB (max value of 2.0 bpm), and 3.0 bpm during running (max value of 4.0 bpm) [225].

#### 7.3.3. Magnetic Field Sensors (Magnetometers)

A magnetic field sensor (also known as magnetometer) can be used to estimate breathing-related thoracic and abdominal movements by recording the corresponding variations in the magnetic vector. Two approaches can be used: (i) the magnetic field sensor can be worn by the subject on the chest (e.g., it can be embedded in straps or clothes) [226] or (ii) the magnetic field sensor can be positioned on the front of the patient’s chest, and a magnet is on the patient’s back [227]. In the first case, the magnetic vector rotates due to the chest movement during the breathing activity and this rotation causes the change of one of the three magnetic vector components [226]. In the second case, the magnetometer detects the strength of the magnetic field (i.e., magnetic flux density) that is inversely proportional to the distance between the sensor and the magnet. Hence, the sensor estimates the position by measuring the intensity of the magnetic field according to the distance between the sensor and the magnet [227]. The use of a passive magnet as a source of magnetic field provides adequate spatial resolution for the specific application and also lower power consumption when compared to other multi-sensors solutions.

When used on humans during regular working activity, good performance was found using the respiratory waveform recorded by a magnetometer to estimate $f_{R}$ values. By the comparison of these values against ones of a reference system (i.e., thermistor), a maximum error of ∼3 bpm was found [226]. Experimental trials on 40 subjects at rest (sitting and standing) and during treadmill exercise demonstrated excellent performance with a MOD of 0.02 s for inspiratory and expiratory time (no data about $f_{R}$) with respect to reference values (i.e., by spirometer) [228].

The magnetometer is highly sensitive to the movement of the body and to external changes of the magnetic field that can occur in unstructured environments. This may negatively affect the quality of the respiratory signal. Magnetic field sensors have also attracted industries for developing smart textiles for respiratory monitoring in clinical settings, as demonstrated by some registered patents [229].

To overcome some of the aforementioned limitations, a 3-axis accelerometer, a 3-axis gyroscope and a 3-axis magnetometer can be integrated into a single device known as inertial measurement unit (IMU). Each IMU provides 3D-acceleration, 3D-magnetic field, and 3D-angular rate [230]. From these quantities, researchers have tried to retrieve respiratory waveforms [230,231]. These data are then combined to provide accurate tridimensional orientation data of the sensor, represented as a quaternion [231]. Computing research on sensors data fusion is growing rapidly because the use of different algorithms may largely affect the outcome of the signal analysis stage, and thus the suitability of IMUs for $f_{R}$ monitoring [230].

### 7.4. Short Summary

Strain and movement sensors may be suitable for monitoring $f_{R}$ because they can be easily integrated into clothes and garments. Piezoresistive, capacitive and inductive sensors can be embedded directly in the textile during garment manufacturing. Differently, accelerometers, gyroscopes and magnetometers can be placed on the garment at specific locations. These sensors are intrinsically unobtrusive when compared to other technologies. Connections (wires) are required to supply the power to the sensor (this applies for strain sensors), but they have high integration in the textile. Sensors are usually placed at the level of the upper chest (at the level of the xiphoid process) and integrated into mechanical supports to give mechanical stability and robustness to the sensor. Strain sensors have exhibited high performance in the monitoring of apnea, SB, QB and FB. One of the main drawbacks of both strain and movement sensors is their sensitivity to movement unrelated to the breathing activity. A possible solution is to develop hybrid solutions based on more sensors to appreciate breathing-unrelated movements and to exclude or reduce their influence on the breathing signal. Piezoresistive sensors can be sensitive to environmental factors changes (i.e., changes in the environmental temperature or humidity may cause an output drift) if not appropriately superficially treated (e.g., incorporated in polymers or protective materials). Movement sensors are generally validated in structured environments and in the clinical scenario (e.g., intensive care unit, geriatrics), while they are seldom employed in the monitoring of athletes especially in outdoor scenarios. All the sensors reviewed in this section allow the recording of the respiratory waveform in the time domain. Additionally, piezoresistive, capacitive, inductive and transthoracic impedance sensors can be used to record respiratory volume after a pre-calibration phase. Given the possibility of integrating multiple sensors in a garment, strain sensors, transthoracic impedance sensors, and movement sensors can be used to analyze the thoracic and abdominal breathing patterns separately. This peculiarity makes these sensors extremely interesting in physiology and biomedical research.

Table 8 summarizes the main metrological properties and characteristics of the movement sensors for $f_{R}$ monitoring, and their field of use.

## 8. Techniques Based on the Modulation of Cardiac Activity

**Figure 10:**
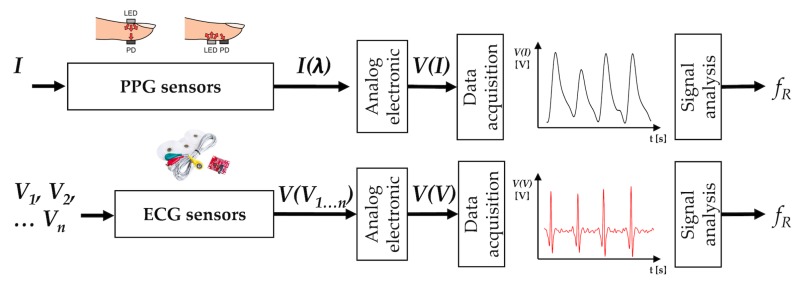
Sensors for recording breathing modulatory effect on cardiac activity: the main elements composing the measuring chain (sensor, analog electronics, data acquisition, post-processing or signal analysis stage) to estimate $f_{R}$. Analog electronic stages vary between different sensors since the output quantities are different. I(λ), $V(V_{1,2,3,\ldots ,n})$ are light intensity and biopotential signals (number 1,2,3,…,n depends on the number of ECG channels), respectively. V(.) is the voltage output. The PPG sensor picture is from [233].

Respiratory activity can be monitored using techniques devoted to recording cardiac activity, in view of the modulatory effect that breathing has on cardiac activity. In particular, electrocardiography, ECG, and photoplethysmography, PPG, can be used to extract $f_{R}$ values (see Figure 10). Both ECG and PPG signals are easily and widely acquired by non-invasive sensors in both healthcare and consumer electronics devices, making them suitable candidates for $f_{R}$ measurement in a range of settings, from hospital to outdoor scenarios [232].

### 8.1. Biopotential Sensors

#### ECG Sensors

ECG is a measure of the electrical activity generated by the action potentials in heart muscle at each heartbeat. ECG signal is typically acquired by measuring the voltage difference between two or more points on the body surface over time [234]. ECG electrode placement is standardized to guarantee accurate recording of the potentials [235,236]. The ECG signal is generally affected by the motion of the electrodes with respect to the heart caused by respiratory-related and -unrelated movements and by changes in the electrical impedance of the thoracic cavity [232]. These phenomena strongly influence the amplitude in the recorded ECG. Specifically, changes in the orientation of the hearth electrical axis related to the electrodes and modification of the thoracic impedance cause the baseline wander and amplitude modulation of the ECG signal [237]. Generally, methods based on changes in the ECG vector orientation are recognized with the EDR acronym (i.e., ECG-derived respiration). The ECG signal is also affected by the frequency modulation caused by the increase of the heart rate during the inspiration phase and its decrease during the expiration phase [238]. Methods based on frequency modulation of the ECG signal are recognized with the RSA acronym (i.e., respiratory sinus arrhythmia) [12]. One or more of these modulations can be used to estimate average values and breath-by-breath values of $f_{R}$, by applying complex algorithms (reviewed in [232,239]) on raw ECG data.

To record biopotential, Ag/AgCl electrodes may be used [236]. However, suitable signals can also be acquired using conductive textiles or polymers integrated into smart clothes [240,241]. Even though the majority of the studies used electrodes positioned on the thorax, promising studies have been published exploring another site (i.e., wrist) to record ECG for $f_{R}$ estimation [242]. The influence of the sensor’s position on the data quality has been explored in [202] by using multi-channel ECG and considering all the pairs of two neighboring electrodes as potential $f_{R}$ sensors. In general, it is not yet clear if multi-channel signals provide better performance compared to one-lead ECG [232]. Given the robustness of the circuits used to record and process the signal, ECG can be incorporated into wearable systems for use with ambulatory patients to identify changes in heart rate and cardiac rhythm.

Breath-by-breath $f_{R}$ analysis during sleep demonstrated an error of 1.8 ± 2.7 bpm when compared to reference data (i.e., by an airflow method) [12]. Experiments on healthy subjects at rest showed a bias of 0.75 ± 2.86 bpm when compared to reference data (i.e., by a wearable system embedding resistive sensors) [243]. The estimation of $f_{R}$ derived from a single-channel ECG wearable device has been demonstrated feasible when tested on 67 subjects during real-life activities (e.g., office, households and sports), with a bias of ∼0.3 ± 3 bpm [244]. Especially during exercise, poor quality of ECG signals can strongly affect the performance of the method for extracting accurate $f_{R}$ values [245]. So far, this method has been mainly used in applications where the ECG was already in use for cardiac monitoring, including intensive care setting and remote monitoring of patients [232].

### 8.2. Light Intensity Sensors

#### PPG Sensors

PPG is an easy and cost-effective optical technique that can be used to measure changes in blood volume over time in a bed of tissue [246]. To record these changes, PPG sensor uses (i) one or more light-emitting diodes, LED, to emit light (with wavelengths between 500 and 600 nm, i.e., the green-yellow region of the visible spectrum) at the tissue [233], and (ii) one photodetector, PD, to record the intensity of the non-absorbed light reflected from the tissue [246]. PPG sensors must be in contact with the tissue. Commonly, the measurement is performed at peripherical sites (i.e., ear, fingers and toes) using pulse oximeter probes that can be easily attached to the skin [233].

The breathing activity modulates the PPG signal in three different ways: (i) amplitude modulation caused by reduced blood stroke volume during the inspiratory phase (resulting by changes in intrathoracic pressure) [247]; (ii) frequency modulation caused by spontaneous increase of heart rate during inspiration phase and related decrease during expiration phase (as in the ECG modulation) [238]; (iii) wander from the signal baseline caused by changes in tissue blood volume (related to intrathoracic pressure changes carried within the arterial tree and vasoconstriction of arteries during inhalation) [248]. Additionally, the respiratory activity affects the PPG pulse wave width [249]. During the inspiratory phase, sympathetic activation stiffens arteries, increasing pulse wave velocity when compared to the expiratory phase. Moreover, intrathoracic pressure changes induced by the respiratory activity can contribute to these stiffness variations [249].

When a PPG sensor is used for measuring $f_{R}$, several factors may be taken into account since the PPG recordings could be affected—among others—by the measuring site (i.e., probe attachment site), the contact force between PPG sensor and site, the subject posture, the ambient temperature [232]. Then, the raw signal is usually corrupted by motion artifacts due to voluntary or involuntary movements of the subject (e.g., during sporting activities) while acquiring the data. Signal processing methods may be used to reduce motion artifacts. Overviews of signal processing techniques that may be used to reduce these artifacts are presented in [233,250]. Additionally, accelerometers or gyroscopes can be used to gather additional information about body site movements and to reduce the motion artifact as presented in [251].

PPG measurement for $f_{R}$ estimation has been tested on patients in clinical settings during procedural sedation and analgesia: in [252] the PPG sensor on the finger showed a limited ability to provide warning signs for a hypoxemic event during the sedation procedure (bias of 2.25 ± 5.41 bpm in the absence of movements). However, a recent multi-center study demonstrated that PPG sensor signals can be used for QB monitoring on healthy subjects and hospitalized patients with a bias always lower than 0.07 ± 3.90 bpm when compared to $f_{R}$ reference data (gathered by capnography [253]). A validation study on 205 children with a wide range of $f_{R}$ (12–50 bpm) during triage (2-min of data collection) demonstrated a bias of 0.9 ± 16.8 bpm [254].

Table 9 summarizes the main metrological properties and characteristics of the sensors for registering cardiac biopotential and light intensity used for $f_{R}$ monitoring, and their field of use.

### 8.3. Short Summary

Undoubtedly, there is a growing interest in the use of ECG and PPG signals for the continuous measurement of $f_{R}$. One of the main reasons is that electrodes for ECG and optical probes for PPG sensors are already used in clinical practice and for home monitoring of heart activity and of oxygen saturation. On the one hand, these sensors are good candidates for continuous $f_{R}$ monitoring since they are characterized by low invasiveness, good integration in wearable systems, low energy consumption, and low cost. On the other hand, their high sensitivity to movements unrelated to breathing may substantially limit their use during exercise. Furthermore, the quality of ECG and PPG signals decreases in the case of a bad electrode or probe adherence to the skin. With the growing use of newly available machine learning, artificial intelligence tools, and advanced signal analysis techniques, respiratory waveforms over time can be retrieved from both raw ECG and PPG signals. A certain number of validation studies have demonstrated good performance of both methods in structured environments (e.g., intensive care unit), while far less studies have been performed outside the clinical scenarios (e.g., during sporting activities). There is a growing interest in this field of research, testified by numerous articles aiming at presenting new techniques and algorithms to be used in the next future for the extraction of respiratory variables and $f_{R}$ values [255].

## 9. Conclusions

In the present review, we have provided a detailed description of the contact-based methods currently available to estimate $f_{R}$. This work was stimulated by the growing interest in monitoring $f_{R}$ during different applications, the difficulty of choosing the appropriate technique to serve different measurement needs, and the paucity of previous reviews on this topic.

Despite the fact that $f_{R}$ is a simple physiological variable, measurement needs may change extensively on the basis of population, activities, environmental factors, costs, device intrusiveness, among other factors. For instance, some techniques (e.g., those based on ECG and PPG recording) may be suitable for measuring $f_{R}$ in clinical settings, but they may not be the preferential choice during sporting activities characterized by motion artifacts. Hence the need for a detailed review that can help understand how to estimate $f_{R}$.

We have identified 7 different methods classified according to the measurand: respiratory airflow, respiratory sound, air temperature, air humidity, air components, chest wall movements, and modulation of cardiac activity. For each method, we have provided a schematic representation of the different sensors, with the description of the elements composing the measuring chain for each sensor. Furthermore, each sensor has been described on the basis of its metrological properties (i.e., sensitivity, response time, output linearity and accuracy), its characteristics (i.e., size, cost, real-time monitoring, measurement intrusiveness, sensitivity to body motion artifacts, influence of environmental factors and presence of wire) and potential applications (i.e., apnea detection, monitoring of SB, QB and FB).

We have also provided a schematic comparison (see Table 10) of the different methods and their suitability in measuring $f_{R}$ in the main fields of application (i.e., clinical settings, occupational settings, and sport and exercise). This schematic comparison can help researchers from various fields and interested readers understand how $f_{R}$ can be monitored for different purposes. Additionally, we have summarized the main advantage and disadvantage taking into account metrological properties and sensor characteristics for each technique used to monitor $f_{R}$.

The proposed classification and methodological approach are expected to guide the choice of techniques for $f_{R}$ estimation and the development of novel respiratory wearables, while stimulating further research.

## Figures and Tables

**Table 1:** Main metrological properties and characteristics related to airflow sensors specifically used for $f_{R}$ measurement. ✓ good to excellent, ∼ sufficient, × poor.

Sensors	Metrological Properties	Sensor Characteristics	Applications
Differential flowmeters	✓ Sensitivity * ✓ Step response time ✓/× Output linearity ** ✓ Accuracy	∼ Sensor size ∼ Cost ✓ Real-time monitoring ∼ Measurement intrusiveness ✓ Sensitivity to body motion artifacts ✓ Influence of environmental factors × Presence of wire	Apnea SB QB FB
Turbine flowmeters	✓ Sensitivity ✓ Step response time ✓ Output linearity ✓ Accuracy	✓/∼ Sensor size * ∼ Cost ✓ Real-time monitoring ∼ Measurement intrusiveness ∼ Sensitivity to body motion artifacts ✓ Influence of environmental factors ∼ Presence of wire	Apnea SB QB FB
Hot wire anemometers	✓ Sensitivity ✓ Step response time × Output linearity ✓ Accuracy	∼ Sensor size ∼ Cost ✓ Real-time monitoring ∼ Measurement intrusiveness × Sensitivity to body motion artifacts ✓ Influence of environmental factors ∼ Presence of wire	Apnea SB QB FB
Fiber-optic sensors	✓ Sensitivity ✓ Step response time ✓ Output linearity ✓ Accuracy	✓ Sensor size × Cost ✓ Real-time monitoring ∼ Measurement intrusiveness × Sensitivity to body motion artifacts ∼ Influence of environmental factors × Presence of wire	Apnea SB QB FB

* it may be adjusted by changing the geometry of the sensor; ** it depends on the kind of sensor.

**Table 2:** Main metrological properties and characteristics of the acoustic sensors used for $f_{R}$ measurement. ✓ good to excellent, ∼ sufficient, × poor.

Sensors	Metrological Properties	Sensor Characteristics	Applications
Microphones	✓ Sensitivity ✓ Step response time ✓/× Output linearity * ✓ Accuracy	✓ Sensor size ✓ Cost ∼ Real-time monitoring ✓ Measurement intrusiveness ✓ Sensitivity to body motion artifacts × Influence of environmental factors ∼ Presence of wire **	Apnea SB QB FB

* it depends on the kind of sensor; ** it depends on the body area on which the sensor is attached (mouth, nose, chest wall).

**Table 3:** Main metrological properties and characteristics of the temperature sensors used for $f_{R}$ measurement. ✓ good to excellent, ∼ sufficient, × poor.

Sensors	Metrological Properties	Sensor Characteristics	Applications
Thermistors	✓ Sensitivity ∼ Step response time ✓ Output linearity ✓ Accuracy	∼ Sensor size ✓ Cost ∼ Real-time monitoring * ∼ Measurement intrusiveness ✓ Sensitivity to body motion artifacts × Influence of environmental factors × Presence of wire	Apnea SB
Thermocouples	✓ Sensitivity ✓ Step response time ✓ Output linearity ✓ Accuracy	✓ Sensor size ∼ Cost ✓ Real-time monitoring ∼ Measurement intrusiveness ✓ Sensitivity to body motion artifacts × Influence of environmental factors × Presence of wire	Apnea SB QB FB
Pyroelectric sensors	✓ Sensitivity ✓ Step response time ✓ Output linearity ✓ Accuracy	✓ Sensor size ∼ Cost ✓ Real-time monitoring ∼ Measurement intrusiveness ✓ Sensitivity to body motion artifacts × Influence of environmental factors × Presence of wire	Apnea SB QB FB
Fiber-optic sensors	✓ Sensitivity ✓ Step response time ✓ Output linearity ✓ Accuracy	✓ Sensor size × Cost ** ✓ Real-time monitoring ∼ Measurement intrusiveness ✓ Sensitivity to body motion artifacts × Influence of environmental factors × Presence of wire	Apnea SB QB FB

* real-time monitoring is not possible for QB, FB; ** it includes the cost of optical spectrum analyzer to power the sensors and record the data.

**Table 4:** Main metrological properties and characteristics of the humidity sensors used for $f_{R}$ measurement. ✓ good to excellent, ∼ sufficient, × poor.

Sensors	Metrological Properties	Sensor Characteristics	Applications
Capacitive sensors	✓ Sensitivity × Step response time * ✓ Output linearity ✓ Accuracy	∼ Sensor size ✓ Cost ✓ Real-time monitoring ∼ Measurement intrusiveness ✓ Sensitivity to body motion artifacts × Influence of environmental factors × Presence of wire	Apnea SB QB **
Resistive sensors	✓ Sensitivity ∼/× Step response time *** ✓ Output linearity ✓ Accuracy	∼ Sensor size ✓ Cost ✓ Real-time monitoring ∼ Measurement intrusiveness ✓ Sensitivity to body motion artifacts × Influence of environmental factors × Presence of wire	Apnea SB QB ** FB **
Nanocrystals and nanoparticles sensors	✓ Sensitivity ✓/∼ Step response time **** ✓ Output linearity ✓ Accuracy	∼ Sensor size ✓ Cost ✓ Real-time monitoring ∼ Measurement intrusiveness ✓ Sensitivity to body motion artifacts × Influence of environmental factors × Presence of wire	Apnea SB QB FB **
Fiber-optic sensors	✓ Sensitivity ✓/∼ Step response time ✓ Output linearity ✓ Accuracy	✓ Sensor size × Cost ✓ Real-time monitoring ∼ Measurement intrusiveness ∼ Sensitivity to body motion artifacts ∼ Influence of environmental factors × Presence of wire	Apnea SB QB FB

* usually some s, but in some cases ∼300 ms; ** it depends on the kind of sensor: QB and FB monitoring is possible only with sensors with short response time; *** in some cases 10 s, but in some cases <50 ms; **** in the worst case 1 s.

**Table 5:** Main metrological properties and characteristics of the air component sensors used for $f_{R}$ measurement. ✓ good to excellent, ∼ sufficient, × poor.

Sensors	Metrological Properties	Sensor Characteristics	Applications
Infrared sensors	✓ Sensitivity ✓ Step response time ✓ Output linearity ✓ Accuracy	∼ Sensor size ∼ Cost ✓ Real-time monitoring ∼ Measurement intrusiveness ✓ Sensitivity to body motion artifacts × Influence of environmental factors × Presence of wire	Apnea SB QB FB
Fiber-optic sensors	✓ Sensitivity ✓ Step response time ✓ Output linearity ✓ Accuracy	✓ Sensor size ∼/× Cost * ✓ Real-time monitoring ∼ Measurement intrusiveness ✓ Sensitivity to body motion artifacts × Influence of environmental factors × Presence of wire	Apnea SB QB FB

* it depends on sensor technology: mid-infrared light fiber-optic sensors are cheaper than long-period fiber gratings and Fabry Perot interferometer.

**Table 6:** Main metrological properties and characteristics of the strain sensors used for $f_{R}$ measurement. ✓ good to excellent, ∼ sufficient, × poor.

Sensors	Metrological Properties	Sensor Characteristics	Applications
Resistive sensors	✓ Sensitivity ✓ Step response time ∼ Output linearity * ✓ Accuracy	✓ Sensor size ✓ Cost ✓ Real-time monitoring ✓/∼ Measurement intrusiveness × Sensitivity to body motion artifacts ∼ Influence of environmental factors ✓ Presence of wire	Apnea SB QB FB
Capacitive sensors	✓ Sensitivity ✓ Step response time ∼ Output linearity ✓ Accuracy	✓ Sensor size ✓ Cost ✓ Real-time monitoring ✓/∼ Measurement intrusiveness × Sensitivity to body motion artifacts ✓ Influence of environmental factors ✓ Presence of wire	Apnea SB QB FB
Inductive sensors	✓ Sensitivity ✓ Step response time ∼ Output linearity ✓ Accuracy	∼ Sensor size (around the chest) ✓ Cost ✓ Real-time monitoring ✓/∼ Measurement intrusiveness ∼ Sensitivity to body motion artifacts ✓ Influence of environmental factors ∼ Presence of wire	Apnea SB QB FB
Fiber-optic sensors	✓ Sensitivity ✓ Step response time ∼ Output linearity ✓ Accuracy	✓ Sensor size ∼ Cost ** ✓ Real-time monitoring ✓/∼ Measurement intrusiveness × Sensitivity to body motion artifacts ✓ Influence of environmental factors ∼ Presence of wire ***	Apnea SB QB FB

* sometimes second order polynomial; ** Low cost in the case of FOSs based on intensity changes; high cost in the case of FBGs because the optical spectrum analyzer is needed; *** Systems based on FBGs require one or more wires to connect the sensors to the optical spectrum analyzer.

**Table 7:** Main metrological properties and characteristics of the impedance sensors used for $f_{R}$ measurement. ✓ good to excellent, ∼ sufficient, × poor.

Sensors	Metrological Properties	Sensor Characteristics	Applications
Impedance sensors	✓ Sensitivity ✓ Step response time ✓ Output linearity ✓ Accuracy	✓ Sensor size ✓ Cost ✓ Real-time monitoring ∼ Measurement intrusiveness × Sensitivity to body motion artifacts ✓ Influence of environmental factors ∼ Presence of wire *	Apnea SB QB FB

* depending on the sensor kind.

**Table 8:** Main metrological properties and characteristics of the movement sensors used for $f_{R}$ measurement. ✓ good to excellent, ∼ sufficient, × poor.

Sensors	Metrological properties	Sensor characteristics	Applications
Accelerometers	✓ Sensitivity ✓ Step response time ✓ Output linearity ✓ Accuracy	✓ Sensor size ✓ Cost ✓ Real-time monitoring ✓ Measurement intrusiveness × Sensitivity to body motion artifacts ✓ Influence of environmental factors ✓ Presence of wire	Apnea SB QB FB
Gyroscopes	✓ Sensitivity ✓ Step response time ✓ Output linearity ✓ Accuracy	✓ Sensor size ✓ Cost ✓ Real-time monitoring ✓ Measurement intrusiveness × Sensitivity to body motion artifacts ✓ Influence of environmental factors ✓ Presence of wire	Apnea SB QB FB
Magnetometers	✓ Sensitivity ✓ Step response time ✓ Output linearity ✓ Accuracy	✓ Sensor size ✓ Cost ✓ Real-time monitoring ✓ Measurement intrusiveness × Sensitivity to body motion artifacts ∼ Influence of environmental factors ✓ Presence of wire	Apnea SB QB FB

**Table 9:** Main metrological properties and characteristics of the biopotential and light intensity sensors used for $f_{R}$ measurement. ✓ good to excellent, ∼ sufficient, × poor.

Sensors	Metrological Properties	Sensor Characteristics	Applications
ECG sensors	✓ Sensitivity ✓ Step response time ✓ Output linearity ∼ Accuracy *	✓ Sensor size ∼ Cost * ✓ Real-time monitoring ∼ Measurement intrusiveness ∼ Sensitivity to body motion artifacts ∼ Influence of environmental factors ∼ Presence of wire **	Apnea SB QB FB
PPG sensors	✓ Sensitivity ✓ Step response time ✓ Output linearity ∼ Accuracy	✓ Sensor size ✓ Cost ✓ Real-time monitoring ✓ Measurement intrusiveness × Sensitivity to body motion artifacts *** ✓ Influence of environmental factors ✓ Presence of wire	Apnea SB QB FB

* it depends on the ECG measuring system; 12-leads medical grade ECG system used in the clinical practice costs over 2000 euro; ** wireless, wearable and implantable ECG measuring systems do not present wires and cables; *** strongly influenced by the body site on which the PPG sensor is positioned (earlobe, finger, etc...).

**Table 10:** Suitability of the different contact-based techniques for monitoring $f_{R}$ in three main fields of application (i.e., clinical settings, occupational settings, and sport and exercise). Each of the three fields has been divided into two parts: part A assesses the suitability in more controlled or structured environments; part B assesses the suitability in less structured or controlled environments. Main advantages and main disadvantages of the different contact-based techniques for monitoring $f_{R}$ are also reported. ✓ good to excellent, ∼ sufficient, × poor.

	CLINICAL	SETTINGS	OCCUPATIONAL	SETTINGS	SPORT AND	EXERCISE		
CONTACT-BASED TECHNIQUE	A	B	A	B	A	B	Main Advantages	Main Disadvantages
Respiratory airflow	✓	∼	✓	∼	✓	∼	Accuracy	Intrusiveness
Respiratory sounds	∼	∼	∼	×	∼	×	Unobtrusiveness	Environmental influence
Air temperature	✓	∼	∼	×	∼	×	Sensitivity	Intrusiveness
Air humidity	∼	∼	∼	×	∼	×	Low sensitivity to motion artifacts	Intrusiveness
Air components	✓	∼	✓	×	∼	×	Accuracy	Intrusiveness
Strain measurements	✓	✓	✓	✓	✓	✓	Unobtrusiveness	Motion artifacts
Impedance measurements	✓	∼	✓	×	∼	×	Unobtrusiveness	Motion artifacts
Movement measurements	∼	∼	∼	∼	∼	×	Unobtrusiveness	Motion artifacts
Biopotential measurements (i.e., ECG)	✓	∼	∼	×	∼	×	Unobtrusiveness	Motion artifacts
Light intensity measurements (i.e., PPG)	✓	∼	∼	×	∼	×	Unobtrusiveness	Motion artifacts

Clinical Settings: A = hospital; B = home/daily activity monitoring. Occupational Settings: A = indoor/structured environment; B = outdoor or unstructured environment. Sport and Exercise: A = indoor/structured environment; B = outdoor or unstructured environment.

## Abbreviations

The following abbreviations are used in this manuscript:

$f_{R}$	Respiratory frequency
bpm	Breaths per minute
SB	Slow breathing
QB	Quiet breathing
FB	Fast breathing
FOS	Fiber-optic sensor
DF	Differential flowmeter
HWA	Hot wire anemometer
FBG	Fiber Bragg Grating
*Q*	Airflow
*P*	Pressure
Δ*P*	Pressure drop
*i*	Current
*R*	Resistance
λ	Wavelength
*I*	Light intensity
*V*	Voltage
FEV1	Forced expiratory volume in the 1st second
FVC	Forced vital capacity
*T*	Temperature
CORSA	Computerized Respiratory Sound Analysis
*C*	Capacitance
*E*	Applied voltage
CO_2_	Carbon dioxide
MOD	Mean of differences
LOA	Limit of agreement
$\lambda_{B}$	Bragg wavelength
RH	Relative humidity
ppm	Parts per million
NDIR	Nondispersive infrared
COPD	Chronic obstructive pulmonary disease
*Z*	Impedance
MEMS	Mechanical and micro-electromechanical system
IMU	Inertial Measurement Unit
PPG	Photoplethysmography
ECG	Electrocardiography
EDR	ECG-derived respiration
RSA	Respiratory sinus arrhythmia
LED	Light-emitting diodes
PD	Photodetector
